# Enzymatic Approach to the Synthesis of Enantiomerically Pure Hydroxy Derivatives of 1,3,5-Triaza-7-phosphaadamantane

**DOI:** 10.1021/acs.joc.0c02586

**Published:** 2021-06-17

**Authors:** Małgorzata Kwiatkowska, Jarosław Błaszczyk, Lesław Sieroń, Piotr Kiełbasiński

**Affiliations:** † Division of Organic Chemistry, Centre of Molecular and Macromolecular Studies, Polish Academy of Sciences, Sienkiewicza 112, 90-363 Łódź, Poland; ‡ Institute of General and Ecological Chemistry, Faculty of Chemistry, Lodz University of Technology, Żeromskiego 116, 90-924 Łódź́, Poland

## Abstract

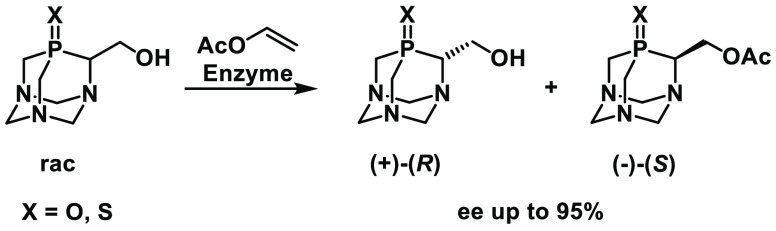

A series of enantiomerically pure derivatives of 6-(1-hydroxyalkyl)-1,3,5-triaza-7-phosphatricyclo[3.3.1.1]decane **5** were successfully synthesized for the first time. A series of hydrolytic enzymes was applied in a stereoselective acetylation performed under kinetic resolution conditions. Although the secondary alcohols: α-aryl-hydroxymethyl-PTA (phosphines) **5b–d′**, PTA-oxides **8b–d′**, and PTA-sulfides **9b–d′** were found to be totally unreactive in the presence of all the enzymes and various conditions applied, the primary alcohols, i.e., the hydroxymethyl derivatives PTA oxide **8a** and PTA sulfide **9a**, were successfully resolved into enantiomers with moderate to good enantioselectivity (up to 95%). The absolute configurations of the products were determined by an X-ray analysis and chemical correlation.

## Introduction

1,3,5-Triaza-7-phosphadamantane (PTA) **1**, whose official IUPAC name is 1,3,5-triaza-7-phosphatricyclo[3.3.1.1]decane, is a water-soluble cage phosphine that has recently received great interest from chemists and has been a subject of several overviews.^1^
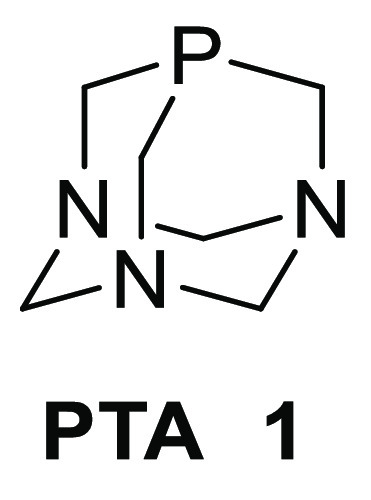


**Figure 1:**
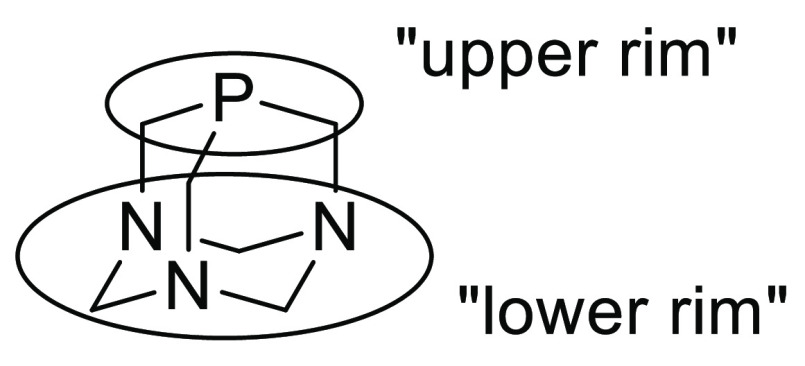
Functionalization of PTA.

Of particular importance are its variously substituted derivatives, including a plethora of metal *P*-complexes exhibiting different catalytic properties. First, PTA can be functionalized either at the “upper rim”, on the phosphorus or carbon atoms, or at the “lower rim”, essentially through quaternization of the nitrogen atoms (Figure 1). The structural modifications introduced in the “lower rim” lead to the changes that are relatively far from the phosphorus atom, being here an important metal coordinating center, and therefore may have a limited influence on the catalytic properties of these compounds. Therefore, a more interesting approach seems to be the introduction of a proper substituent at the methylene group adjacent to the phosphorus atom of PTA (“upper rim”). Moreover, this should result in the formation of chiral PTA analogues with the stereogenic carbon atom located close to the metal coordinating center on phosphorus.

**Scheme 1:**
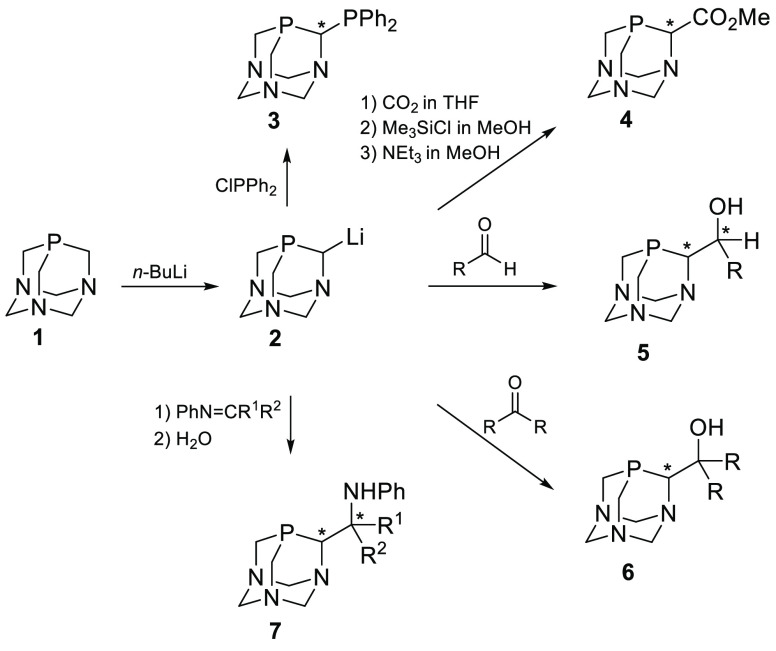
Reaction of PTA-Li **2** with Electrophiles

Such a functionalization was presented in several papers, of which the most important was the treatment of the reactive synthon PTA-Li **2** with various electrophiles, e.g., with dichlorophenylphosphine,^2^ carbon dioxide, aldehydes, ketones, or imines (Scheme 1).^3^

As mentioned above all the products bear a stereogenic center at the carbon atom α to the phosphorus atom. Additionally, the reaction of PTA-Li with prochiral electrophiles, e.g., aldehydes or imines, leads to the diastereomeric products, having two stereogenic centers, **5** and **7**, respectively, which could be separated by repeated crystallization. However, in all cases only racemic products were described. Surprisingly, no attempts to obtain enantiomerically pure (or at least enriched) derivatives of PTA have been made thus far. Therefore, we have decided to develop a methodology which would enable one to produce chiral nonracemic derivatives of PTA, which would then serve as chiral, water-soluble ligands or catalysts.

**Scheme 2:**
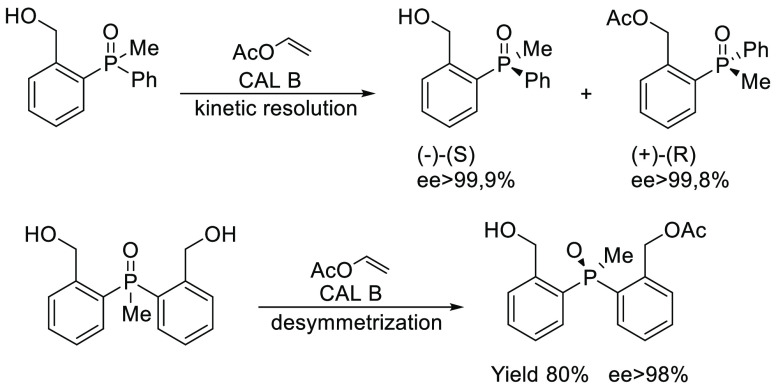
Enzymatic Synthesis of Enantiomerically Pure Phosphine Oxides

In the frame of the research carried out in our laboratory, enzymes were intensely used for the synthesis of optically active heteroorganic compounds, particularly those containing a stereogenic center located on a heteroatom–phosphorus^4,5^ or sulfur.^6,7^ Some time ago, we reported the synthesis of phosphine oxide precursors of chiral bidentate and tridentate phosphorus catalysts via hydrolytic enzyme-promoted kinetic resolution of racemic *P*-chiral or desymmetrization of *P*-prochiral phosphine oxides, which allowed us to obtain the desired products in enantiopure forms (Scheme 2).^8^ For a review see ref (9).

**Scheme 3:**
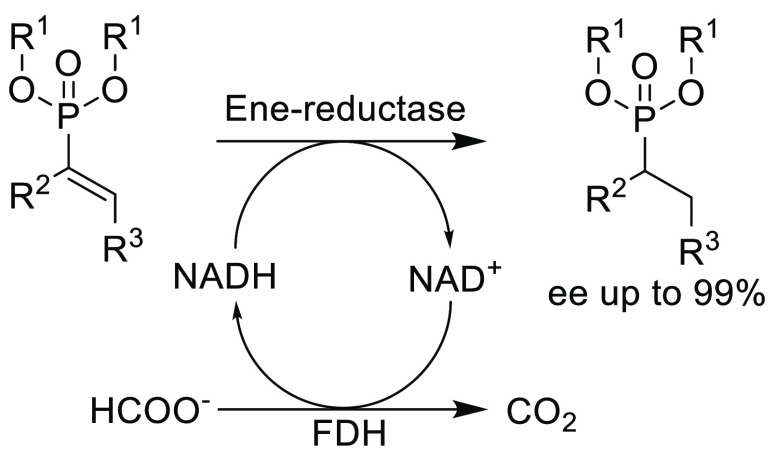
Asymmetric Bioreduction of β-Activated Vinylphosphonates

Another approach, this time concerning the synthesis of *C*-chiral organophosphorus compounds, comprised asymmetric bioreduction of β-activated vinylphosphonates using ene-reductases, which resulted in the formation of almost enantiomerically pure products (Scheme 3).^10^ Similar satisfactory results were obtained using *Mucor circinelloides* whole cells as a source of the desired enzymes.^11^

Taking advantage of these results, we decided to check whether the enzymatic approach would be suitable for our purposes.

## Results and Discussion

### Synthesis of Substrates

**Scheme 4:**
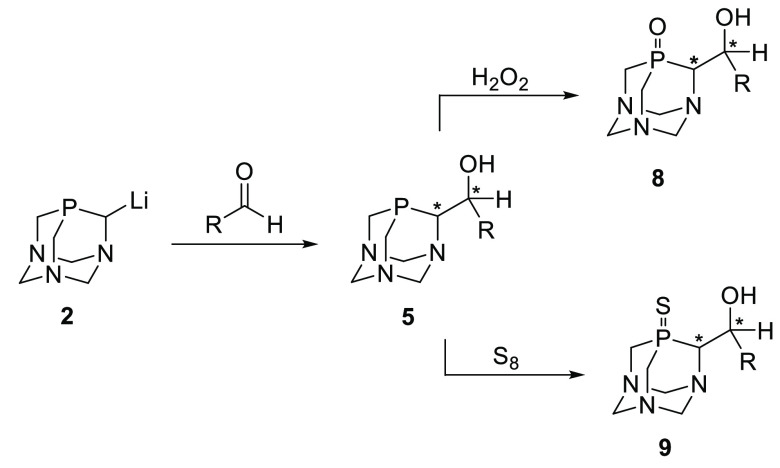
Synthesis of Substrates for Enzymatic Transformations

We began our studies with the synthesis of a number of racemic 1-hydroxyalkyl derivatives of PTA using PTA-Li **2** as substrate. Its reactions with aryl carbaldehydes were performed according to the literature procedures,^3a,3c^ and the racemic diastereomers of secondary alcohols **5b–d′** were separated by a column chromatography using dichloromethane/methanol or ethyl acetate/methanol as solvents. The reaction of PTA-Li with paraformaldehyde was performed for the first time in a similar way to give the hitherto unknown racemic 1-hydroxymethyl derivative **5a** (R = H). Due to a susceptibility to oxidation of the phosphines obtained, they were transformed into the corresponding phosphine oxides **8** and phosphine sulfides **9** (Scheme 4), and the latter were used in the ensuing transformations. The results are collected in Table 1.

**Table 1:** Racemic Substrates for Enzymatic Transformations^a^

		phosphine **5**		phosphine oxide **8**		phosphine sulfide **9**		
symbol	R	yield (%)	^31^P NMR (δ)	yield (%)	^31^P NMR (δ)	yield (%)	^31^P NMR (δ)	remarks
**a**	H	45	–102.2	24	–1.9	30	–15.5	
**b**	Ph	15	–106.5	40	–5.1	41	–17.3	diastereomers
**b′**	Ph	21	–103.0	41	–3.7	42	–13.5	
**c**	*p-*BrC_6_H_4_	16	–106.7	31	–5.0	32	–17.4	diastereomers
**c′**	*p-*BrC_6_H_4_	6	–102.4	13	–4.0	14	–13.3	
**d**	*p*-Me_2_NC_6_H_4_	17	–106.2	30	–5.0	32	–17.0	diastereomers
**d′**	*p*-Me_2_NC_6_H_4_	7	–102.7	10	–3.8	11	–13.0	

a Conditions: 3.2 mmol of PTA, 3.3 mmol of *n*-BuLi, 3.5 mmol of aldehyde, THF (20 mL) under argon then an excess of 35% aqueous solution of H_2_O_2_ or elemental sulfur (2 equiv)

### Attempts at the Enzymatic Kinetic Resolution of Racemic Substrates

**Scheme 5:**
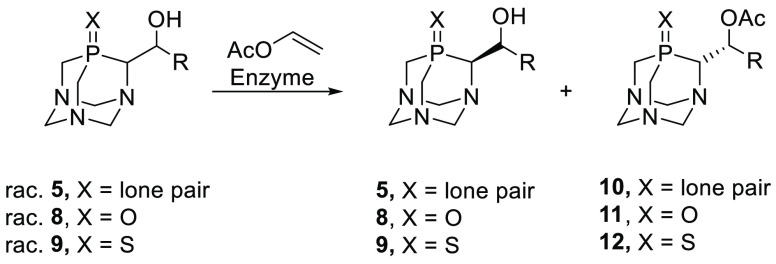
Enzymatic Kinetic Resolution of Racemic Substrates **5–9**

All of the substrates were subjected to enzyme-catalyzed hydroxyl group acetylation under kinetic resolution conditions (Scheme 5). The enzyme set consisted of a variety of commonly accessible hydrolytic enzymes, particularly lipases.

First, we started with the secondary alcohols **5b–d′**, **8b–d′**, and **9b–d′** in the hope of getting more interesting enantiomeric products due to the simultaneous presence of two stereogenic centers in their easily separable racemic diastereomers. However, the great disappointment for us was a total lack of reactivity of these substrates, in spite of the use of a broad variety of enzymes (for the list of enzymes see Table 2 and footnotes) and conditions. What was particularly surprising was the fact that, according to the literature data, the scope of secondary alcohols transformed by enzymes in this way is practically unlimited!^12^

**Table 2:** Enzymatic Kinetic Resolution of Racemic Substrates

				recovered substrate			product, after hydrolysis			
substrate	enzyme^a^	solvent	time (day)	yield (%)	ee^b^ (%)	abs conf	yield (%)	ee^b^ (%)	abs conf	*E*^c^
**9a**	CAL-B	CH_2_Cl_2_	2	14	95	*R*	44	64	*S*	16
**9a**	CRL	CH_2_Cl_2_	2	32	54	*R*	59	12	*S*	2
**9a**	TL	CH_2_Cl_2_	8	32	72	*R*	58	38	*S*	4.5
**9a**	Mix: CAL-B, PFL, PS, CRL, CCL	CH_2_Cl_2_	7	48	68	*R*	47	72	*S*	13
**9a**	Mix: PPL, MJ, AK, LPL, AH	CH_2_Cl_2_	10	74	6	*R*	25	30	*S*	2
**9a**	PCP	CH_2_Cl_2_	7							
**9a**	WGL	CH_2_Cl_2_	7							
**9a**	PA	CH_2_Cl_2_	7							
**9a**	MJ	CH_2_Cl_2_	7							
**9a**	LPL	CH_2_Cl_2_	7							
**9a**	PFL	CH_2_Cl_2_	7							
**9a**	CCL	CH_2_Cl_2_	7							
**8a**	TL	CH_2_Cl_2_	8	34	44	*R*	36	32	*S*	3
**8a**	Mix: PFL, CAL-B, PS, CRL, CCL	CH_2_Cl_2_	7	41	33	*R*	54	30	*S*	2.5
**8a**	CAL-B	CH_2_Cl_2_	2	59	23	*R*	32	27	*S*	2
**8a**	CRL	CH_2_Cl_2_	2	53	24	*R*	27	32	*S*	2.5
**8a**	PFL	CH_2_Cl_2_	22	70	7	*R*	20	14	*S*	1.5
**8a**	CCL	CH_2_Cl_2_	22	46	12	*R*	19	19	*S*	2
**8a**	CRL	C_6_H_12_	4	38	10	*R*	13	7	*S*	1
**8a**	CRL	MeCN	4	49	10	*R*	1	9	*S*	1.5
**8a**	CRL	Et_2_O	4	47	10	*R*	15	11	*S*	1.5
**8a**	CRL	toluene	4	45	18	*R*	15	20	*S*	2

a Enzymes: CAL-B, *Candida antarctica* lipase; PFL, *Pseudomonas fluorescens* lipase; PS, *Pseudomonas species* lipase; CRL, *Candida rugose* lipase; CCL, *Candida cylindracea* lipase; TL, lipase from *Pseudomonas stutzeri*; PPL, *Porcine pancreas* lipase; MJ, *Mucor javanicus* lipase; AK, lipase AK (AMANO); LPL, lipoprotein lipase; PCP, papain from *Carica papaya*; WGL, *wheat germ* lipase; AH, lipase AH (AMANO); PA, penicillin amidase.

b The ee was determined using HPLC with chiral columns: AS-H, *n*-hexane (*i*-PrOH/EtOH 4:1) 75:25, Fl. 0.5 mL/min for P=S derivatives **9a** and OD-H, *n*-hexane:(MeOH/EtOH 1:1) 75:25, Fl. 0.5 mL/min for P=O derivatives **8a**

c The enantiomer ratio *E* = ln[1 – *c*(1 + ee_p_)]/ln[1 – *c*(1 – ee_p_)], *c* = ee_s_/(ee_s_ + ee_p_).^13^

As we assumed that in our case the lack of reactivity could be due to the presence of two spatially demanding substituents (PTA and an aryl group), we decided to resort to the use of less hindered molecules. The substrates of choice were the 1-hydroxymethyl derivatives of PTA **5a**, **8a**, and **9a**. Preliminary experiments using phosphine **5a** indicated that it was impossible to avoid its substantial oxidation to the phosphine oxide **8a**. Therefore, the ensuing experiments were performed using PTA-oxide **8a** and PTA-sulfide **9a** as substrates for the acetylation performed as shown in Scheme 5. The results are collected in Table 2.

Inspection of Table 2 clearly shows that substrates **8a** and **9a** were recognized and transformed by several enzymes, and the corresponding 1-acetoxymethyl derivatives of PTA **11a** and **12a**, respectively, were formed and could be separated from the unreacted substrates. The enantiomeric excess of the substrate was determined using chiral HPLC. The same procedure was used to determine the ee of the products **11a** and **12a**; however, they had to be first hydrolyzed to the hydroxymethyl derivatives **8a** and **9a**. Generally, better results were obtained in the case of hydroxymethyl PTA-sulfide **9a** than hydroxymethyl PTA-oxide **8a**, both in terms of yield and enantiomeric excess. The best result was achieved when the racemic compound **9a** was acetylated in the presence of CAL B in dichloromethane.

### Determination of the Absolute Configuration of **9a**: X-ray Analysis

**Figure 2:**
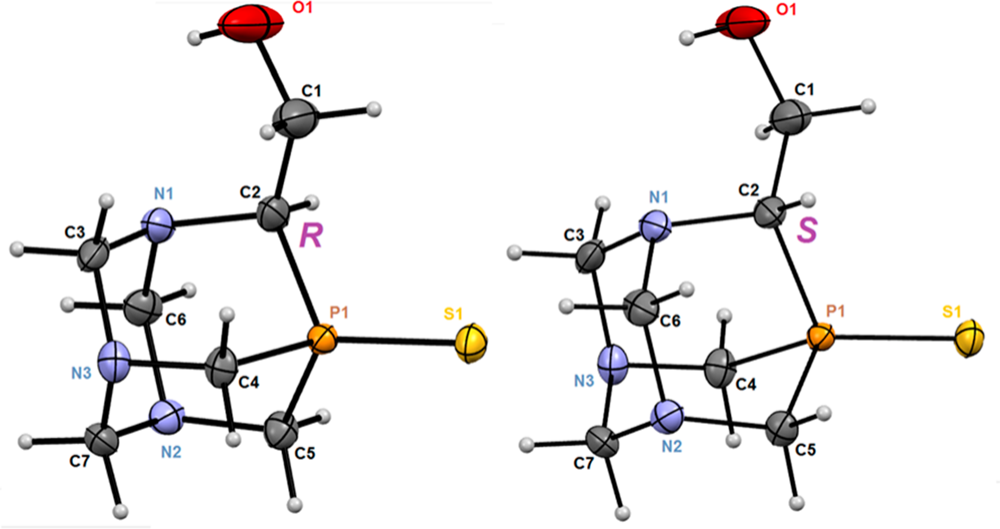
Ellipsoidal view (ORTEP) of the enantiomeric molecule (*R*)-**9a** (left) and (*S*)-**9a** (right), showing the single molecules present in the asymmetric units, the atom numbering scheme, and the absolute configuration of the substituents at the carbon atom C2. Ellipsoids are drawn with 50% probability level.

**Figure 3:**
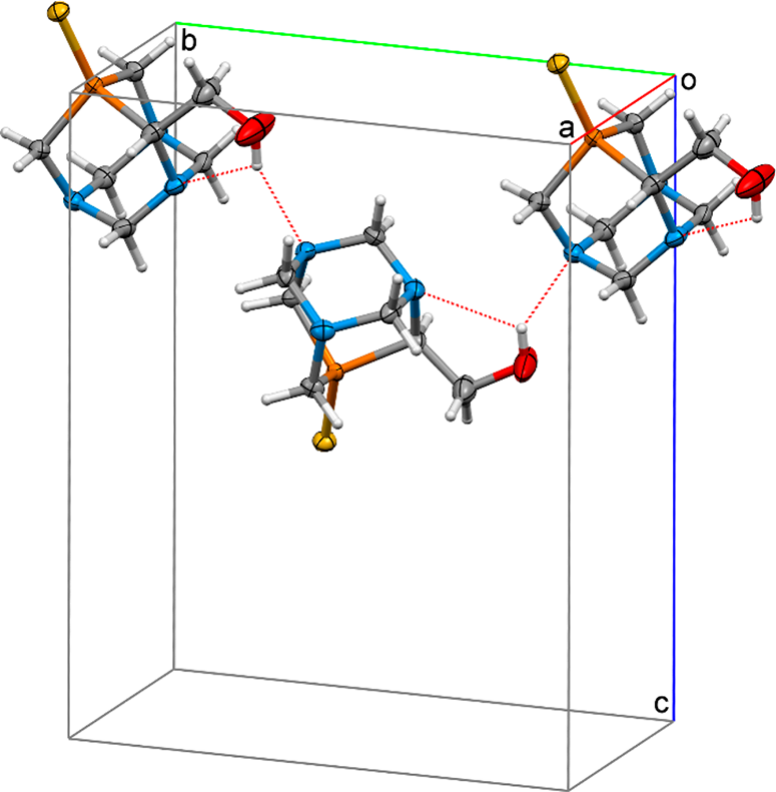
Fragment of the packing of molecules in the crystal of compound **9a** with a marked chain of intermolecular hydrogen bonds extending along the axis *b* of the unit cell.

To perform an X-ray analysis, the enantiomerically enriched hydroxymethyl PTA-sulfide derivatives obtained were crystallized from methanol to give crystals of pure enantiomers of **9a**. Both (*R*) and (*S*) enantiomers crystallize in the noncentrosymmetric space group *P*2(1)2(1)2(1) of the orthorhombic system. Asymmetric parts of the unit cells of both compounds are composed of individual molecules (Figure 2). Both enantiomers are isostructural: enantiomer (+)-(*R*)-**9a** ([α]_389_ +3.25, *c* = 1.3, MeOH) and the opposite enantiomer (−)-(*S*)-**9a** ([α]_389_ −3.24, *c* = 0.9, MeOH) have nearly identical geometry and conformation. The enantiomer (*R*) and the mirror image of the enantiomer (*S*) superimpose very well, and the rmsd value is less than 0.001 Å. Of particular interest, the rotation of the O1–C1 bond around the C1–C2 bond in both structures is nearly identical, as shown by the values of the torsion angle P1–C2–C1–O1, equal to 175.2° in (*R*)-**9a** and 174.6° in (*S*)-**9a**. The molecule **9a** has an urotropine-type structure in which one nitrogen atom has been replaced by a phosphorus atom and is bonded to a sulfur atom. All P–C bond lengths in both structures are equal within experimental error with an average distance of 1.831 Å. The location of the hydroxyl group appears to be determined and supported by the weak intramolecular hydrogen bond O1–H(O1)···N1 with an O···N distance of 2.870 Å and O–H···N angle of 115° (calculated as the average of both enantiomers). The crystal packing system is built of molecules connected by intermolecular hydrogen bonds O1–H(O1)···N2 (1 – *x*, *y* – 1/2, 1/2 – *z*), with the distances H(O1)···N2 equal to 2.085 Å (calculated as the average of both enantiomers), forming one-dimensional chains running along *b* axis of the unit cell (Figure 3). The N3 and O1 atoms are donors of weak C–H···N and C–H···O intermolecular contacts assembling a 3D supramolecular network (Table 3).

**Table 3:** Hydrogen-Bonding Geometry for **9a** (Å/deg)

	H···A	D···A	D–H···A	symmetry
O1–H1···N1	2.41/2.41	2.867(4)/2.872(3)	115/115	
O1–H1···N2	2.09/2.08	2.800(4)/2.798(3)	143/143	1 – *x*, 1/2 + *y*, 3/2 – *z*
C1–H2···N3	2.56/2.55	3.534(5)/3.531(4)	166/167	1 + *x*, *y*, *z*
C1–H11···O1	2.20/2.23	3.151(7)/3.153(5)	162/162	–1/2 + *x*, 3/2 – *y*, 1 – *z*

The absolute configuration of each enantiomer was determined as a result of the analysis of anomalous X-ray scattering. The Flack *x* parameters determined for the (*R*)-**9a** and (*S*)-**9a** enantiomers have values of 0.005(10) and −0.012(8), respectively.

CIF files containing complete information on both studied enantiomers of 6-(1-hydroxymethyl)(1,3,5-triaza-7-phosphoadamantane) P-sulfide **9a** have been deposited with the Cambridge Crystallographic Data Centre, and the reference codes are CCDC 1842806 for enantiomer (*R*)-**9a** and CCDC 1842807 for enantiomer (*S*)-**9a**.

### Determination of the Absolute Configuration of **8a**: Chemical Correlation

**Scheme 6:**
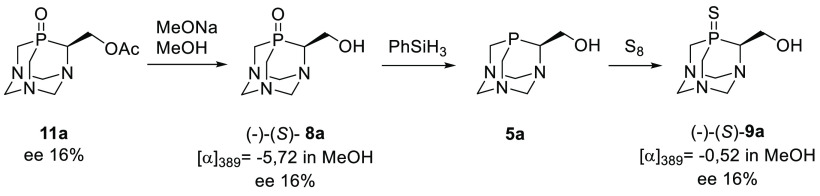
Determination of the Absolute Configuration of **8a** by a Chemical Correlation

To determine the absolute configuration of hydroxy derivative of PTA(O) **8a**, whose pure enantiomer could not be obtained by crystallization, a chemical correlation was performed. To this end, the enantiomerically enriched acetyl derivative **11a**, i.e., the product of the enzymatic acetylation of **8a** under the kinetic resolution, was chosen and transformed into **9a** (Scheme 6). First, compound **11a** was hydrolyzed to the corresponding nonracemic derivative **8a** whose ee was determined using chiral HPLC to be 16% ([α]_389_ −5.72, c = 0.7, MeOH). In the next step, the P=O moiety was reduced using phenylsilane to form phosphine derivative **5a** (based on the ^31^P NMR analysis of the crude reaction mixture). Then elemental sulfur was added to produce the sulfur analogue (*S*)-**9a** ([α]_389_ −0.52, *c* = 0.4, MeOH). The latter exhibited the same enantiomeric excess as the starting **8a**, and its prevailing enantiomer corresponded directly to the prevailing enantiomer of **8a**. Since in all the transformations the stereogenic center on the α-carbon atom remained untouched, it may be concluded that in both cases the same enantiomer (*S*)-**8a** of the substrate was recognized by the active center of the enzyme and converted into the corresponding acetyl derivative (Scheme 6).

## Conclusions

We have applied a series of hydrolytic enzymes in a stereoselective acetylation of 1-hydroxyalkyl derivatives of PTA under the kinetic resolution conditions. Although the secondary alcohols **5b–d′**, **8b–d′**, and **9b–d′** turned out to be totally unreactive in the presence of all the enzymes and under various conditions applied, the hydroxymethyl derivatives **8a** and **9a** were successfully resolved into enantiomers with moderate to good enantioselectivity. The absolute configuration of pure enantiomers of **9a** was determined by an X-ray analysis, while that of **8a** was determined by a chemical correlation. This is the first successful synthesis of enantiomerically pure derivatives of PTA, and it paves the way for the synthesis of enantiomeric organocatalysts and ligands for metal complexes which can be used as chiral catalysts in asymmetric synthesis, e.g., the reactions which have so far been performed only in an achiral manner. The results of the transformations and functionalization of the compounds obtained, including formation of metal complexes, as well as their application in asymmetric synthesis will be published elsewhere.

## Experimental Section

### General Methods

The synthesized products were purified by column chromatography on Merck 60 silica gel (0.063–0.200 mm) or preparative plate chromatography using Merck 60 F_254_ silica gel plate (2.5 mm). TLC was performed on a Merck 60 F_254_ silica gel plate (0.25 mm). Solvents were dried using general procedures and distilled prior to use. The NMR spectra were recorded in CDCl_3_, D_2_O, or MeOD with a Bruker AV 200 spectrometer. The chemical shifts (δ) are expressed in ppm, and the coupling constants (*J*) are given in hertz. Mass spectra were recorded with a Finnigan MAT95 Voyager Elite spectrometer. Optical rotations were measured on a Perkin–Elmer 241 MC polarimeter. HPLC analysis were made using Varian Pro Star 210 instrument using column with chiral filling Chiralcel OD-H or Chiralpak AS-H. The enzymes are defined in Table 2.

The X-ray data for compounds (*S*)-**9a** and (*R*)-**9a** were collected with a Bruker APEX-II CCD diffractometer at room temperature using CuKα radiation, and the crystal structures and absolute configurations were determined with SHELXL-97.^14^

### Synthesis of the Racemic Substrate 6-(1-Hydroxy(1-aryl)alkyl)(1,3,5-triaza-7-phosphaadamantane) **5b**–**d**

These compounds were synthesized according to the literature procedures described in ref (3a,3c).

### Synthesis of the Racemic Substrate 6-(1-Hydroxymethyl)(1,3,5-triaza-7-phosphaadamantane) (**5a**)

To a suspension of PTA (0.5 g, 3.185 mmol) in THF (15 mL) under nitrogen was added *n*-BuLi (1.1 eq, 1.25 mL 3.503 mmol; 2.7 M solution in *n*-heptane) dropwise at room temperature. The mixture was stirred for 3 h at room temperature, and then paraformaldehyde (1.0 g, 35 mmol) was added in portions. The stirring was continued at room temperature, and the progress of the reaction was monitored by ^31^P NMR of the crude reaction mixture. Then methanol (10 mL) was added, and solvents were evaporated. The crude product was used for further transformations (yield of crude product as a white solid: 0.268 g, 45%). ^31^P{^1^H} NMR (CD_3_COCD_3_, 81 MHz): δ −102.2. ^1^H NMR (CD_3_COCD_3_, 200 MHz): δ 4.81–4.65 (m, 1H, CH), 4.58–3.48 (m, 12H, CH_2_).

### Synthesis of the Racemic Substrate 6-(1-Hydroxymethyl)(1,3,5-triaza-7-phosphaadamantane) *P*-Oxide (**8a**)

To obtain the oxide derivatives of PTA, **8a**, to the suspension of crude **5a** (0.275 g, 1.471 mmol) in distilled water (20 mL) was added an excess of 35% aqueous solution of H_2_O_2_ (3 mmol). After 30 min, ^31^P NMR analysis of the crude reaction mixture showed the formation of corresponding oxide compounds. Crude reaction product was purified by column chromatography using ethyl acetate–methanol in gradient with the addition of triethylamine (0.03% vol) to give pure **8a** as a white solid (isolated yield: 0.072 g, 24%). ^31^P{^1^H} NMR (CD_3_OD, 202 MHz): δ −1.9. ^1^H NMR (CD_3_OD, 500 MHz): δ 4.77–4.64 (m, 1H, CH), 4.52–3.81 (m, 12H, CH_2_). ^13^C{^1^H} NMR (CD_3_OD, 126 MHz): δ 73.6 (d, ^3^*J*_CP_ = 6 Hz, CH_2_N), 71.5 (d, ^3^*J*_CP_ = 9 Hz, CH_2_N), 65.9 (d, ^3^*J*_CP_ = 12 Hz, CH_2_N), 65.2 (d, ^1^*J*_CP_ = 2 Hz, CHP), 59.5 (s, CH_2_OH), 54.6 (d, ^1^*J*_CP_ = 51 Hz, CH_2_P), 52.6 (d, ^1^*J*_CP_ = 50 Hz, CH_2_P). MS (CI) *m*/*z*: [M + H]^+^ 204; HRMS (TOF MS ES+) *m*/*z*: [M + H]^+^ Calcd for C_7_H_15_N_3_O_2_P 204.0902; Found 204.0900.

### Synthesis of the Racemic Substrate 6-(1-Hydroxymethyl)(1,3,5-triaza-7-phosphaadamantane) *P*-Sulfide (**9a**)

To obtain the sulfur derivative of PTA **9a**, to the suspension of crude **5a** (0.275 g, 1.471 mmol) in dichloromethane (20 mL) under nitrogen elemental sulfur (0.094 g, 2.947 mmol) was added. The mixture was stirred at room temperature until the substrate disappeared, which was found by ^31^P NMR. Then the reaction mixture was filtered through Celite and the solvent was evaporated. The crude reaction mixture was purified by column chromatography using ethyl acetate–methanol in gradient with the addition of triethylamine (0.03% vol) to give pure **9a** as a white solid (isolated yield: 0.097 g, 30%). ^31^P{^1^H} NMR (CD_3_OD, 81 MHz): δ −15.5. ^1^H NMR (CD_3_OD, 500 MHz): δ 4.75–4.69 (m, 1H, CH), 4.59–3.83 (m, 12H, CH_2_). ^13^C{^1^H} NMR (CD_3_OD, 126 MHz): δ 74.1 (d, ^3^*J*_CP_ = 6 Hz, CH_2_N), 71.6 (d, ^3^*J*_CP_ = 9 Hz, CH_2_N), 66.5 (d, ^1^*J*_CP_ = 37 Hz, CHP), 65.4 (d, ^3^*J*_CP_ = 12 Hz, CH_2_N), 58.8 (d, ^2^*J*_CP_ = 4 Hz, CH_2_OH), 58.2 (d, ^1^*J*_CP_ = 37 Hz, CH_2_P), 55.5 (d, ^1^*J*_CP_ = 35 Hz, CH_2_P). MS (CI) *m*/*z*: [M + H]^+^ 220. HRMS (TOF MS ES+) *m*/*z*: [M + H]^+^ Calcd for C_7_H_15_N_3_OPS 220.0673; Found 220.0674.

### General Procedure for the Enzymatic Acetylation of the Racemic Substrates **8a** and **9a**

To a solution of the racemic substrate **8a** or **9a** (20.3 mg for **8a** or 21.9 mg for **9a**, 0.1 mmol) in a solvent (5 mL) were added vinyl acetate (0.5 mL) and an enzyme (5 mg). The whole mixture was stirred at room temperature. The conversion degree was determined by ^31^P NMR. Then the enzyme was filtered off and the solvent evaporated. The crude reaction mixture was separated by column chromatography using ethyl acetate–methanol in gradient from 20:1 to 1:1 with the addition of triethylamine (0.03% vol) as eluent to give the pure enantiomerically enriched substrates **8a** (yield: 6.9–14.2 mg; 34–70%) or **9a** (yield: 3.1–16.2 mg; 14–74%) and *O-*acetyl products **11a** (yield: 0.2–7.8 mg; 1–32%), or **12a** (yield: 6.5–15.4 mg; 25– 59%) as a white solids. Then the acetyl products were hydrolyzed using catalytic amount of MeONa in MeOH (3 mL) to give pure enantiomerically enriched opposite enantiomers of the substrates **8a** or **9a**, respectively. The enantiomeric excesses of the alcohols obtained were determined by HPLC using column with chiral filling: OD-H for P=O derivatives **8a** (*n*-hexane/(MeOH/EtOH 1:1) 75:25%; Fl. 0.5 mL/min; wavelength 224 nm; 18.9 min for (*R*) enantiomer and 20.1 min for (*S*) enantiomer) and AS-H for P = S derivatives **9a** (*n*-hexane/(*i*-PrOH/EtOH 4:1) 75%: 25%; Fl. 0.5 mL/min; wavelength 224 nm; 36.4 min for (*S*) enantiomer and 40.8 min for (*R*) enantiomer). The results are collected in Table 2.

#### Acetoxymethyl Oxide **11a**

^31^P{^1^H} NMR (CDCl_3_, 202 MHz): δ −3.3. ^1^H NMR (CD_3_OD, 500 MHz): δ 4.92–4.86 (m, 1H, CH), 4.58–3.85 (m, 12H, CH_2_), 2.09 (s, 3H, Me). ^13^C{^1^H} NMR (CD_3_OD, 126 MHz): δ 179.0 (s, C = O), 73.6 (d, ^3^*J*_CP_ = 6 Hz, CH_2_N), 71.5 (d, ^3^*J*_CP_ = 9 Hz, CH_2_N), 65.9 (d, ^3^*J*_CP_ = 12 Hz, CH_2_N), 65.2 (d, ^1^*J*_CP_ = 52 Hz, CHP), 59.5 (s, CH_2_O), 54.6 (d, ^1^*J*_CP_ = 51 Hz, CH_2_P), 52.6 (d, ^1^*J*_CP_ = 50 Hz, CH_2_P), 22.7 (s, CH_3_). MS (CI) *m*/*z*: [M + H]^+^ 246; HRMS (TOF MS ES+) *m*/*z*: [M + H]^+^ Calcd for C_9_H_17_N_3_O_3_P 246.1008; Found 246.1007.

#### Acetoxymethyl Sulfide **12a**

^31^P{^1^H} NMR (CDCl_3_, 81 MHz): δ −18.3. ^1^H NMR (CD_3_OD, 500 MHz): δ 5.09–4.91 (m, 1H, CH), 4.60–3.85 (m, 12H, CH_2_), 2.07 (s, 3H, Me). ^13^C{^1^H} NMR (CD_3_OD, 126 MHz): δ 170.9 (s, C = O), 73.8 (d, ^3^*J*_CP_ = 6 Hz, CH_2_N), 71.5 (d, ^3^*J*_CP_ = 9 Hz, CH_2_N), 65.2 (d, ^3^*J*_CP_ = 11 Hz, CH_2_N), 63.8 (d, ^1^*J*_CP_ = 36 Hz, CHP), 60.5 (d, ^2^*J*_CP_ = 7 Hz, CH_2_O), 58.1 (d, ^1^*J*_CP_ = 37 Hz, CH_2_P), 55.3 (d, ^1^*J*_CP_ = 35 Hz, CH_2_P), 19.4 (s, CH_3_). MS (CI) *m*/*z*: [M + H]^+^ 262; HRMS (TOF MS ES+) *m*/*z*: [M + H]^+^ Calcd for C_9_H_17_N_3_O_2_PS 262.0779; Found 262.0780.

### Chemical Correlation

To the enantiomerically enriched acetyl derivative of PTA(O) **11a** (7.3 mg, 0.029 mmol) in methanol (2 mL) was added a catalytic amount of MeONa (2 M in methanol). The reaction mixture was stirred at room temperature until the substrate disappeared (TLC ethyl acetate/methanol 5:1 or ^31^P NMR). Then the solvent was evaporated, and the product was purified by column chromatography using ethyl acetate: methanol in gradient (from 20:1 to 1:5) to give with quantitative yield hydroxyl derivative **8a** (whose ee was determined using chiral HPLC column with chiral filling: OD-H (*n*-hexane/(MeOH: /EtOH 1:1) 75:25; Fl. 0.5 mL/min; wavelength 224 nm; 18.9 min for (*R*) enantiomer and 20.1 min for (*S*) enantiomer). In the next step, to the solution of substrate **8a** (6.1 mg, 0.029 mmol) in toluene (2 mL) under nitrogen atmosphere was added phenylsilane (0.097 g, 30 equiv, 0.09 mmol). The reaction mixture was refluxed using an oil bath until the substrate disappeared (based on ^31^P NMR of the crude reaction mixture). After cooling to room temperature, elemental sulfur (1.9 mg, 2 equiv, 0.060 mmol) was added. The reaction mixture was stirred overnight, and then the precipitate was filtered off through Celite and washed with methanol. The solvents were evaporated, and the crude product was purified by column chromatography using ethyl acetate/methanol in a gradient with the addition of triethylamine (0.03% vol) as eluent to afford sulfur derivative **9a** (isolated yield after two steps: 5 mg, 76%). The enantiomeric excess was determined using chiral HPLC (AS-H (*n*-hexane/(*i*-PrOH/EtOH 4:1) 75:25%; Fl. 0.5 mL/min; wavelength 224 nm; 36.4 min for (*S*) enantiomer and 40.8 min for (*R*) enantiomer).

## Supporting Information Available

Detailed crystallographic data, HPLC chromatograms, and NMR spectra of new compounds (PDF)

## Accession Codes

CCDC 1842806–1842807 contain the supplementary crystallographic data for this paper. These data can be obtained free of charge via www.ccdc.cam.ac.uk/data_request/cif, or by emailing data_request@ccdc.cam.ac.uk, or by contacting The Cambridge Crystallographic Data Centre, 12 Union Road, Cambridge CB2 1EZ, UK; fax: +44 1223 336033.

## Dedication

Dedicated to Professor Grzegorz Mlostoń, University of Łódź, on the occasion of his 70th birthday.
