# Supplementary material for: Enzymatic Approach to the Synthesis of Enantiomerically Pure Hydroxy Derivatives of 1,3,5-Triaza-7-phosphaadamantane
Source: J Org Chem. 2021 Jun 17;86(13):8556–62. doi: 10.1021/acs.joc.0c02586 (PMC8279493; doi:10.1021/acs.joc.0c02586)

SUPPORTING INFORMATION

Enzymatic Approach to the Synthesis of Enantiomerically Pure Hydroxy Derivatives of 1,3,5-Triaza-7-phosphaadamantane (PTA)

Małgorzata Kwiatkowska,[a*] Jarosław Błaszczyk,[a] Lesław Sieroń,[b]
and Piotr Kiełbasiński [a]

[a] Division of Organic Chemistry, Centre of Molecular and Macromolecular Studies, Polish Academy of Sciences, Sienkiewicza 112, 90-363 Łódź, Poland
[b] Institute of General and Ecological Chemistry, Faculty of Chemistry, Lodz University of Technology, Żeromskiego 116, 90-924 Łódź, Poland

E-mail: gosia@cbmm.lodz.pl

Table of contents

1. Crystallographic data S-2
2. HPLC chromatograms S-6
3. NMR spectra S-10

1. Crystallographic data

The crystals of both enantiomers of 6-hydroxymethyl(1,3,5-triaza-7-phosphoadamantane) P-sulfide: (+)-(*R*)-**9a** ($[\alpha]_{389}$ = +3.25 in MeOH), and the opposite enantiomer (-)-(*S*)-**9a** ($[\alpha]_{389}$ = -3.24 in MeOH), suitable for X-ray analysis, were obtained by recrystallization from methanol. For both enantiomers the single-crystal X-ray diffraction data were collected at 100K by the ω-scan technique on a XtaLAB Synergy, Dualflex, Pilatus 300K diffractometer using PhotonJet micro-focus X-ray Source CuKα (λ = 1.54184 Å) as a source of radiation. Absorption corrections were based on the indexing of the crystal faces using the CrysAlis PRO program[S1]. The structure was solved by direct methods with SHELXT-2018/2[S2] and followed by successive Fourier and difference Fourier syntheses and refined by full-matrix least-squares on F2 using the SHELXL-2018/3 program[S2]. All non-hydrogen atoms in the complex were refined with anisotropic thermal parameters. The hydrogen atoms were placed in calculated positions and refined isotropically with a riding model except for those bound to oxygen atoms, which were initially located in a difference Fourier map and refined using the AFIX 147 command. The absolute configurations of (*R*)-**9a** and (*S*)-**9a** were determined from anomalous scattering by calculating the x Flack parameter of 0.005(10) and -0.012(8), respectively[S3]. The *Coot* and *Mercury* programs were used for model building and structure visualization[S4, S5].

For (*R*)-**9a**, the Flack parameter was x = 0.005(10). For all data, the final wR^2 was 0.0695, R_1 = 0.0264, S = 1.138, max residual density = 0.285 eÅ^{-3} (see Table S1). The calculated parameters for inverted structure, i.e. with assumed opposite (incorrect) chirality, were: $x_{(inv)}$ = 1.00(4), $wR^2_{(inv)}$ = 0.0974, and $R_{1(inv)}$ = 0.0380.

For (*S*)-**9a**, the Flack parameter was x = -0.012(8). For all data, the final wR^2 was 0.0584, R_1 = 0.0223, S = 1.149, max residual density = 0.253 eÅ^{-3} (see Table S1). The calculated parameters for inverted structure, i.e. with assumed opposite (incorrect) chirality, were: $x_{(inv)}$ = 1.04(3), $wR^2_{(inv)}$ = 0.0916, and $R_{1(inv)}$ = 0.0360.

Table S1.

Crystal data and refinement details for enantiomers (*R)* and (*S)* of 6-hydroxymethyl-(1,3,5-triaza-7-phosphoadamantane) P-sulfide (**9a**).

**Compound**	(*R*)-**9a**	(*S*)-**9a**
Molecular formula	$C_7H_{14}N_3OPS$	$C_7H_{14}N_3OPS$
CCDC accession number	CCDC 1842806	CCDC 1842807
Formula weight	219.24	219.24
Temperature, K	100	100
Wavelength, Å	1.54178 (CuK_{α})	1.54178 (CuK_{α})
Crystallographic system	Orthorhombic	Orthorhombic
Space group	$P2_12_12_1$	$P2_12_12_1$
F(000)	464	464
a, Å	6.07424(9)	6.09772(4)
b, Å	11.29821(12)	11.30980(7)
c, Å	14.07285(18)	14.10243(8)
Volume, $Å^3$	965.79(2)	972.560(10)
Z	4	4
Crystal size, mm	0.39, 0.23, 0.10	0.34, 0.14, 0.08
Calculated density, g cm^{-3}	1.508	1.497
Absorption coefficient, mm^{-1}	4.271	4.241
θ range for data collection, °	6.29 - 66.55	5.01 - 66.60
Limiting indices (*h*, *k*, *l*)	-6/7, -13/13, -16/16	-7/6, -13/13, -16/16
Reflections collected / unique	10567 / 1683	23662 / 1722
Data / restraints / parameters	1683 / 0 / 121	1722 / 0 / 120
R_{int}	0.0324	0.0292
x Flack parameter	0.005(10)	-0.012(8)
Goodness-of-fit on F^2	1.138	1.149
R_1 [$F^2 > 2\sigma(F^2)$]	0.0264	0.0223
R_1 (all data)	0.0264	0.0223

wR^2 [$F^2 > 2\sigma(F^2)$]	0.0695	0.0584
wR^2 (F^2)	0.0695	0.0584
Max difference peak, hole, eÅ^{-3}	0.285, -0.368	0.253, -0.263

Weighting details:

w(**1**) = $1/[\sigma^2(F_o^2) + (0.0296P)^2 + 0.7567P]$ where $P = (F_o^2 + 2F_c^2)/3$

w(**2**) = $1/[\sigma^2(F_o^2) + (0.0224P)^2 + 0.6232P]$ where $P = (F_o^2 + 2F_c^2)/3$

CIF files containing complete information on both studied enantiomers of 6-hydroxymethyl-(1,3,5-triaza-7-phosphoadamantane) P-sulfide **9a** have been deposited with the Cambridge Crystallographic Data Centre, and the reference codes are: CCDC 1842806 for enantiomer (*R*)-**9a**, and CCDC 1842807 for enantiomer (*S*)-**9a**. These files can be obtained free of charge from the CCDC, from the following web site: www.ccdc.cam.ac.uk/data_request/cif.

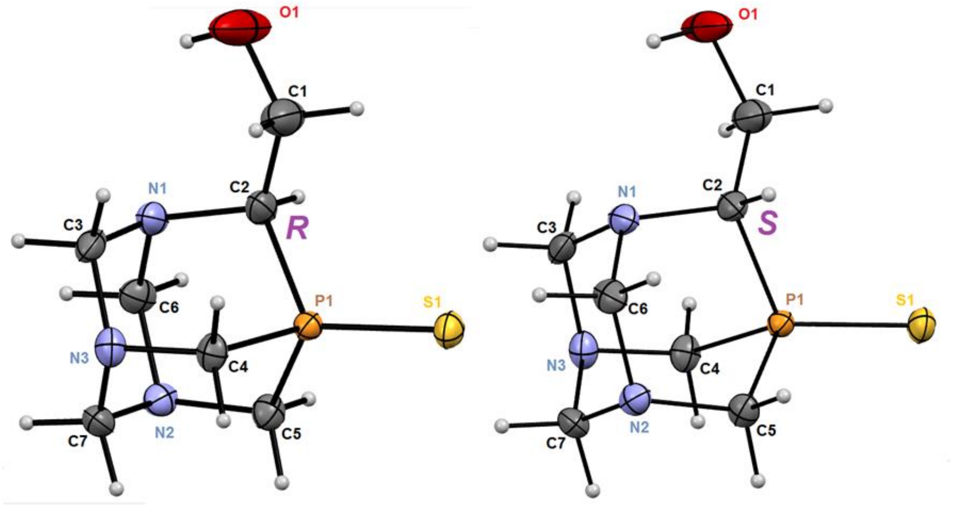

Figure S1. Ellipsoidal view (ORTEP) of the enantiomeric molecule (*R*)-**9a** (left) and (*S*)-**9a** (right), showing the single molecules present in the asymmetric units, the atom numbering scheme, and the absolute configuration of the substituents at the carbon atom C2. Ellipsoids are drawn with 50% probability level.

References

[S1]. Rigaku OD (2015). CrysAlis PRO. Rigaku Oxford Diffraction Ltd, Yarnton, Oxfordshire, England.

[S2]. Sheldrick, G.M. Crystal Structure Refinement with SHELXL. *Acta Cryst.* **2015**, *C71*, 3-8.

[S3]. Flack, H.D.; Shmueli, U. The Mean-Square Friedel Intensity Difference in P1 with a Centrosymmetric Substructure. *Acta Cryst.* **2007**, *A63*, 257-265.

[S4]. *Coot Version 0.6.1.* Emsley, P.; Lohkamp, B.; Scott, W.G.; Cowtan, K. (2010). Features and development of Coot. *Acta Cryst.* **2010**, *D66*, 486-501.

[S5]. *Mercury CSD 3.0.* Macrae, C.F.; Bruno, I.J.; Chisholm, J.A.; Edgington, P.R.; McCabe, P.; Pidcock, E.; Rodriguez-Monge, L.; Taylor, R.; van de Streek, J.; and Wood, P.A. New Features for the Visualization and Investigation of Crystal Structures. *J. Appl. Cryst.* **2008**, *41*, 466-470.

2. HPLC chromatograms

HPLC analysis were made using column with chiral filling: Chiralcel OD-H for P=O derivatives **8a** (Hexane : (MeOH : EtOH 1:1) 75% : 25%; Fl. 0.5 mL/min; wavelength 224 nm; 18.9 min for (*R*) enantiomer and 20.1 min for (*S*) enantiomer) and Chiralpak AS-H for P=S derivatives **9a** (Hexane : (iPrOH : EtOH 4:1) 75% : 25%; Fl. 0.5 mL/min; wavelength 224 nm; 36.4 min for (*S*) enantiomer and 40.8 min for (*R*) enantiomer).

a) PTA(O)-CH_2OH **8a** racemate

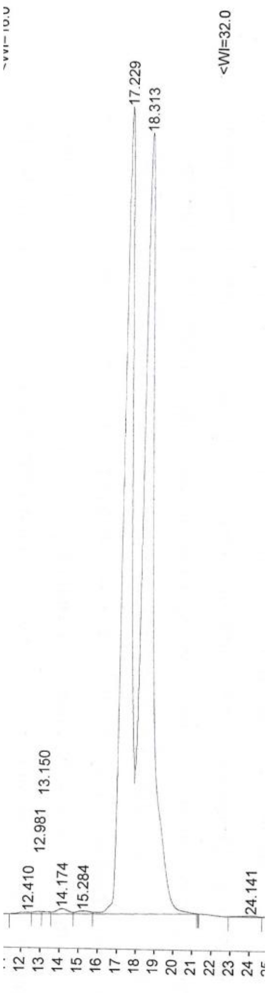

b) PTA(O)-CH_2OH **8a** ee 32%

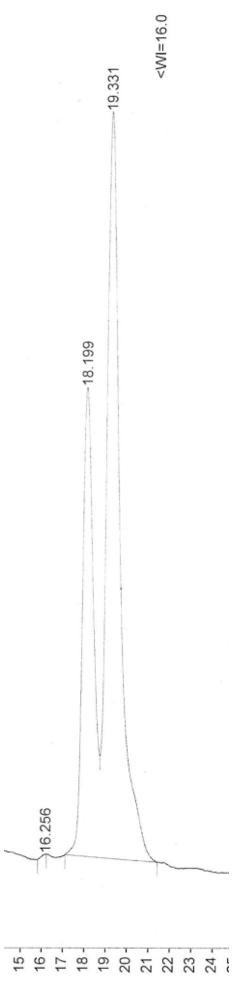

c) PTA(S)-CH_2OH **9a** racemate

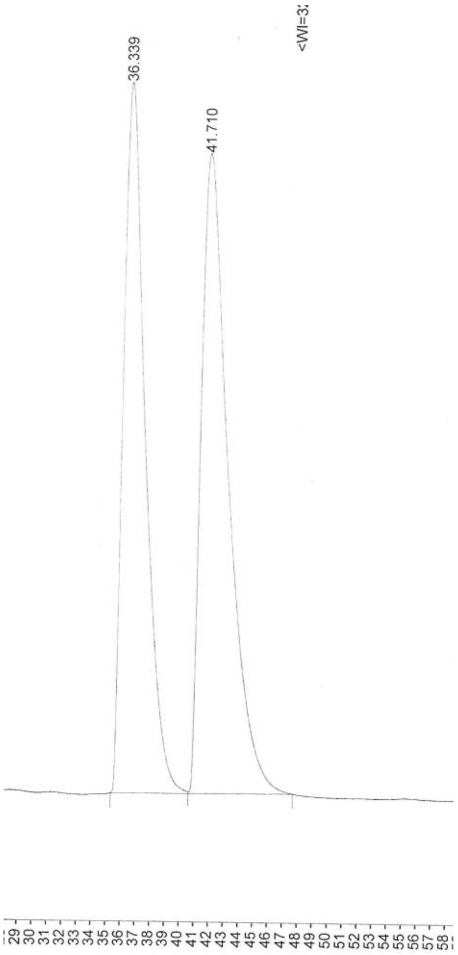

d) PTA(S)-CH_2OH **9a** ee 86%

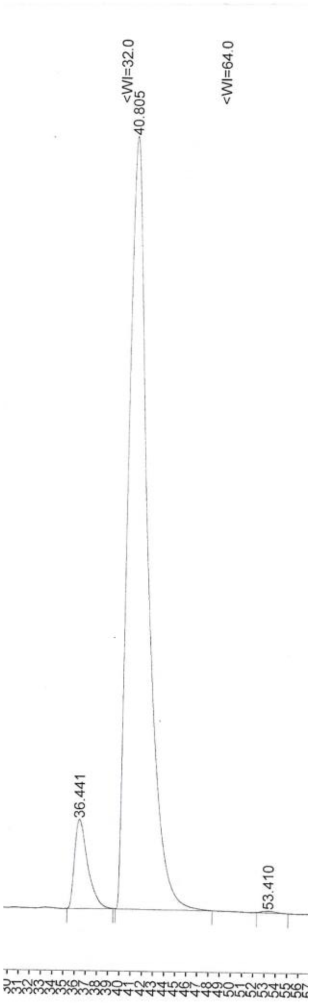

3. NMR spectra

PTA-CH_2OH **5a**

$^{31}P\{^1H\}$ NMR (CD_3COCD_3, 81 MHz)

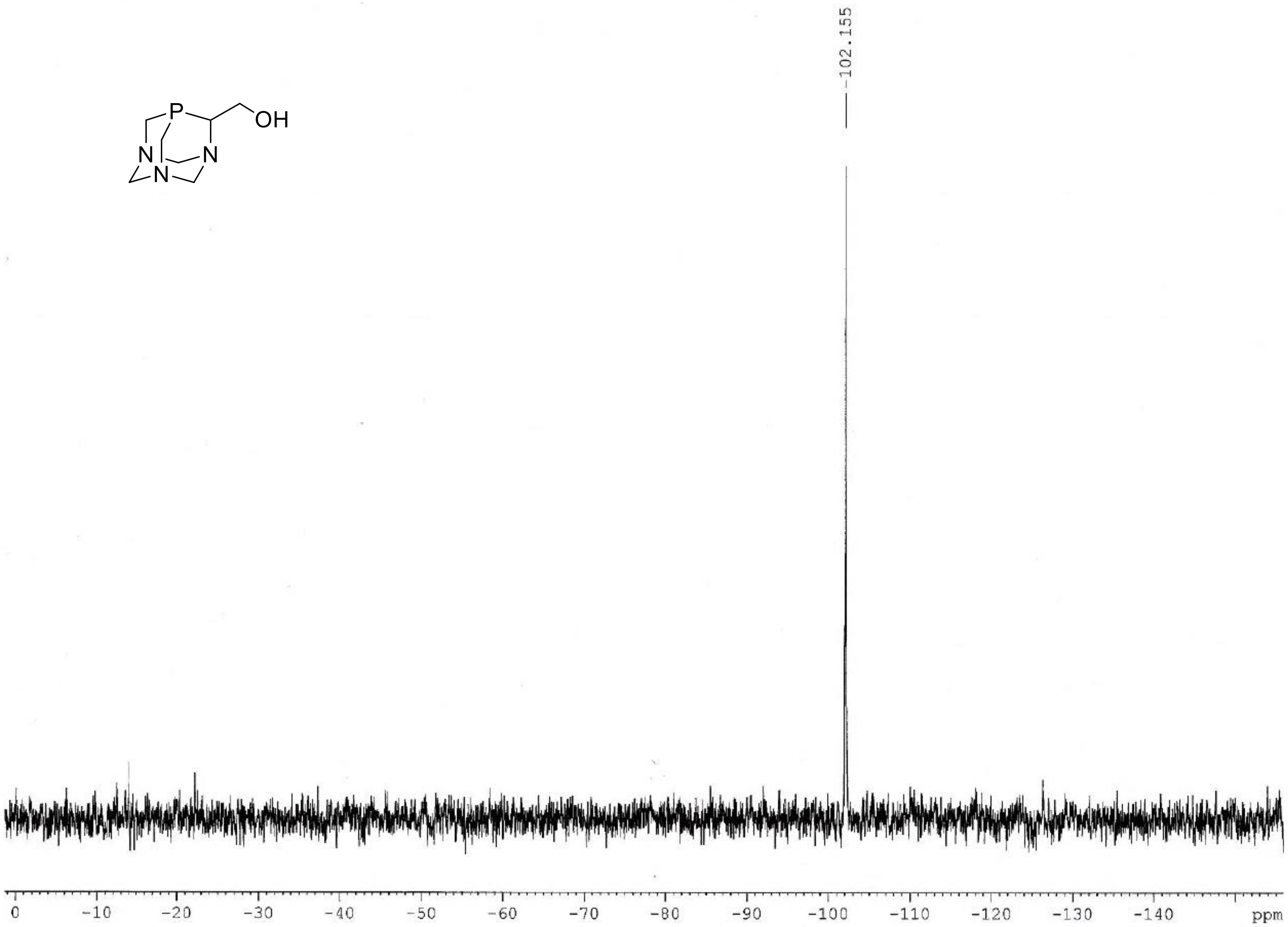

PTA-CH_2OH **5a**

^1H NMR (CD_3COCD_3, 200 MHz)

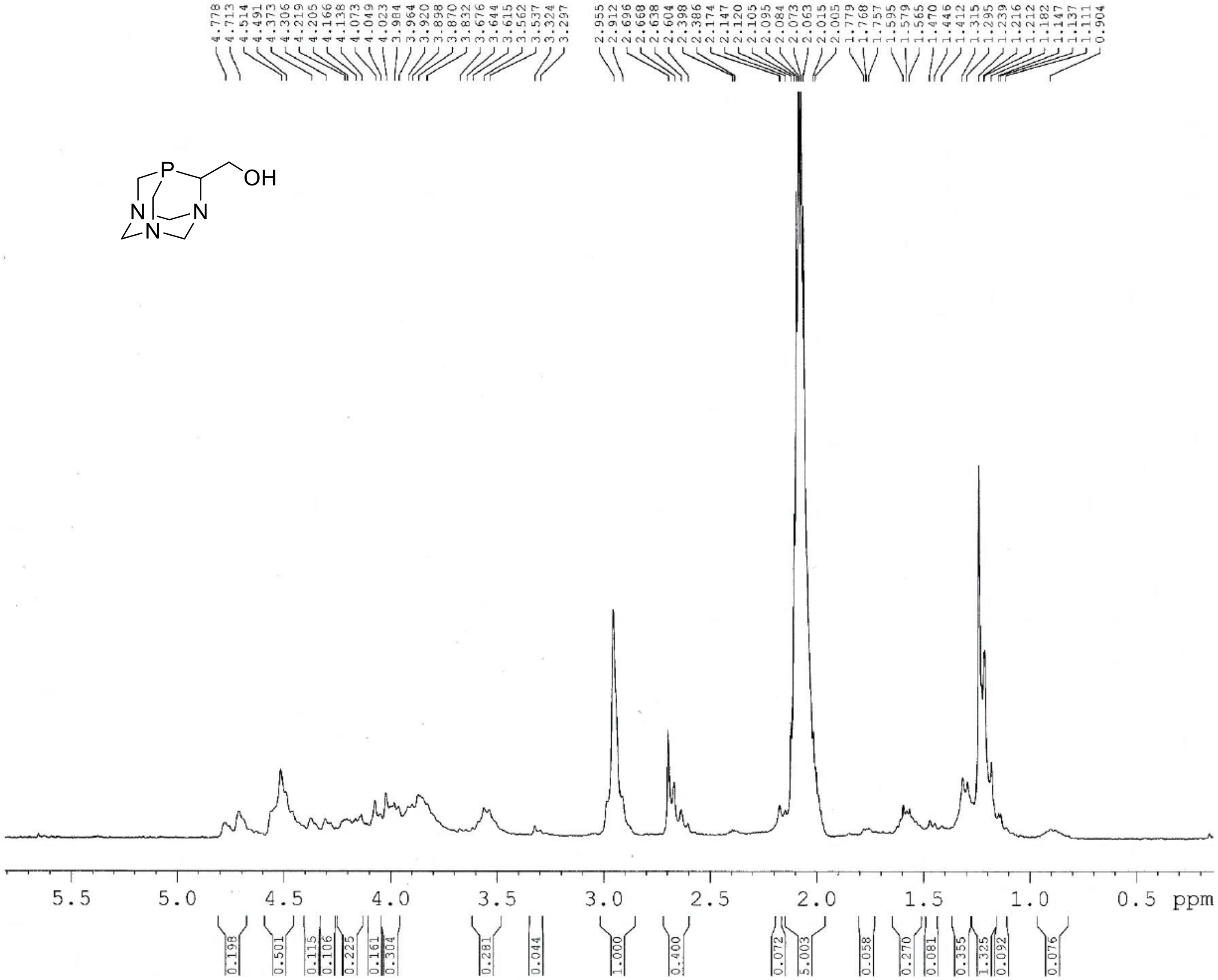

PTA(O)-CH_2OH **8a**

$^{31}P\{^1H\}$ NMR (CD_3OD, 202 MHz)

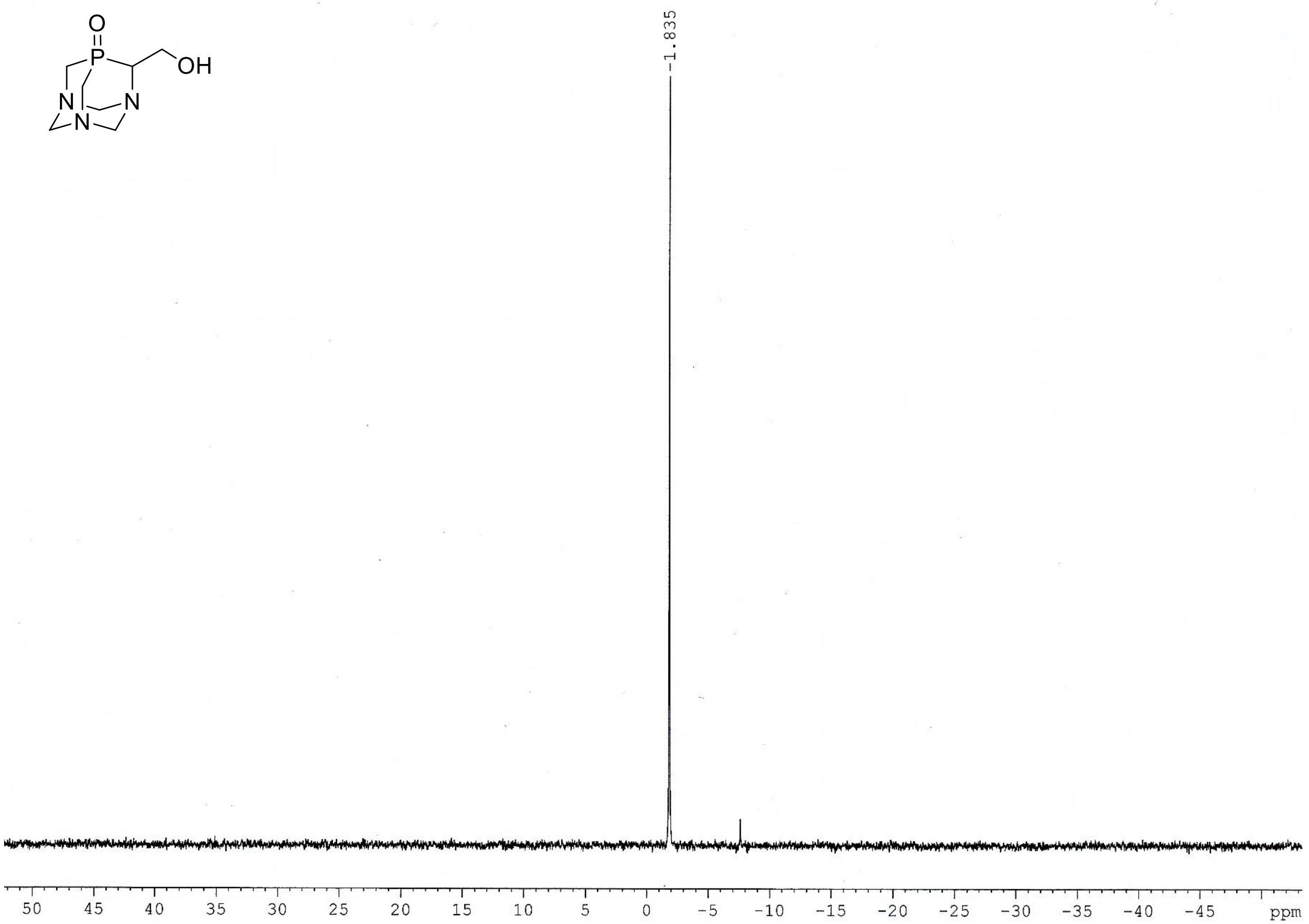

PTA(O)-CH_2OH **8a**

^{1}H NMR (CD_3OD, 500 MHz)

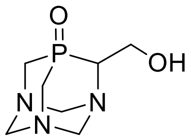

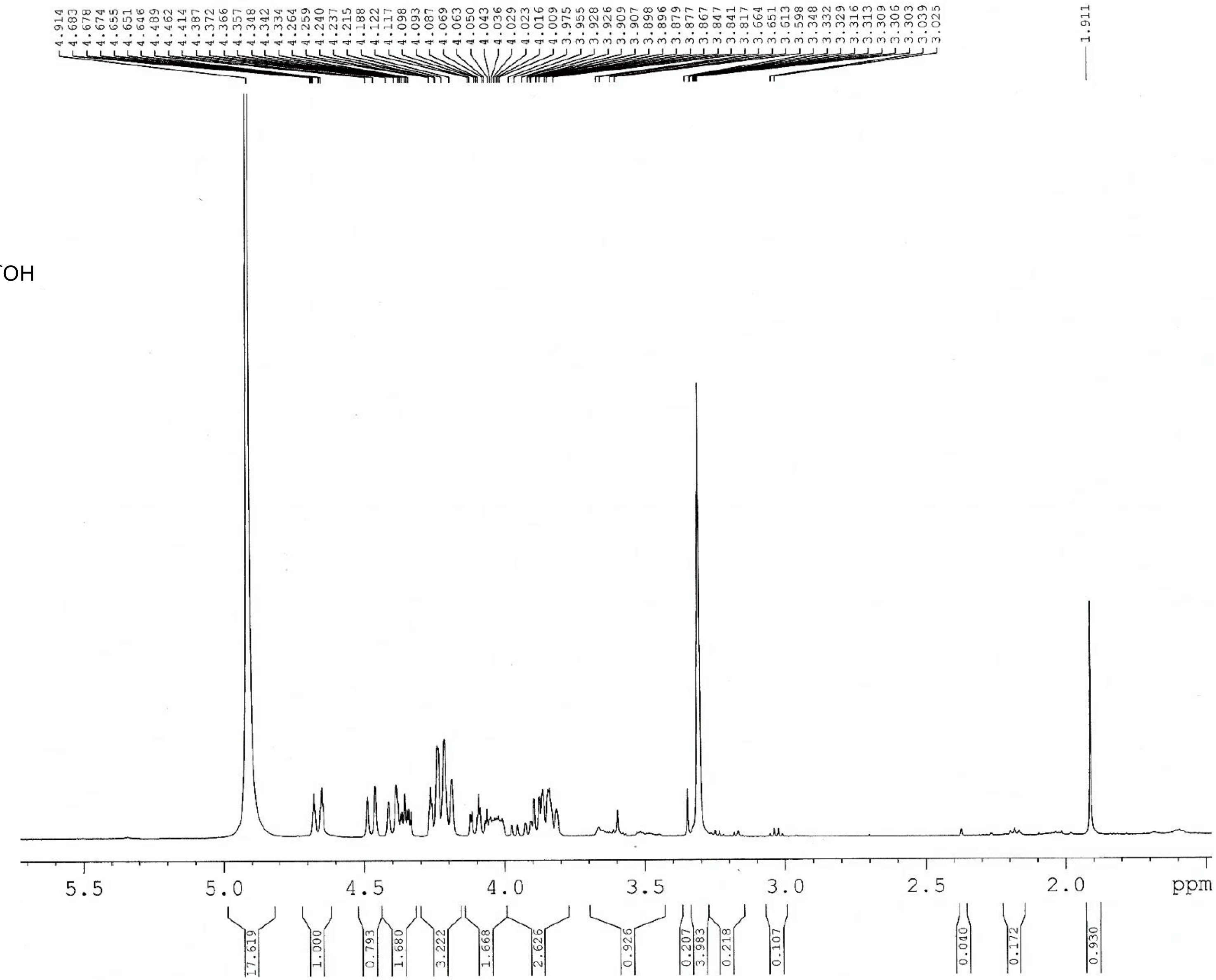

PTA(O)-CH_2OH **8a**

$^{13}C\{^1H\}$ NMR (CD_3OD, 126 MHz)

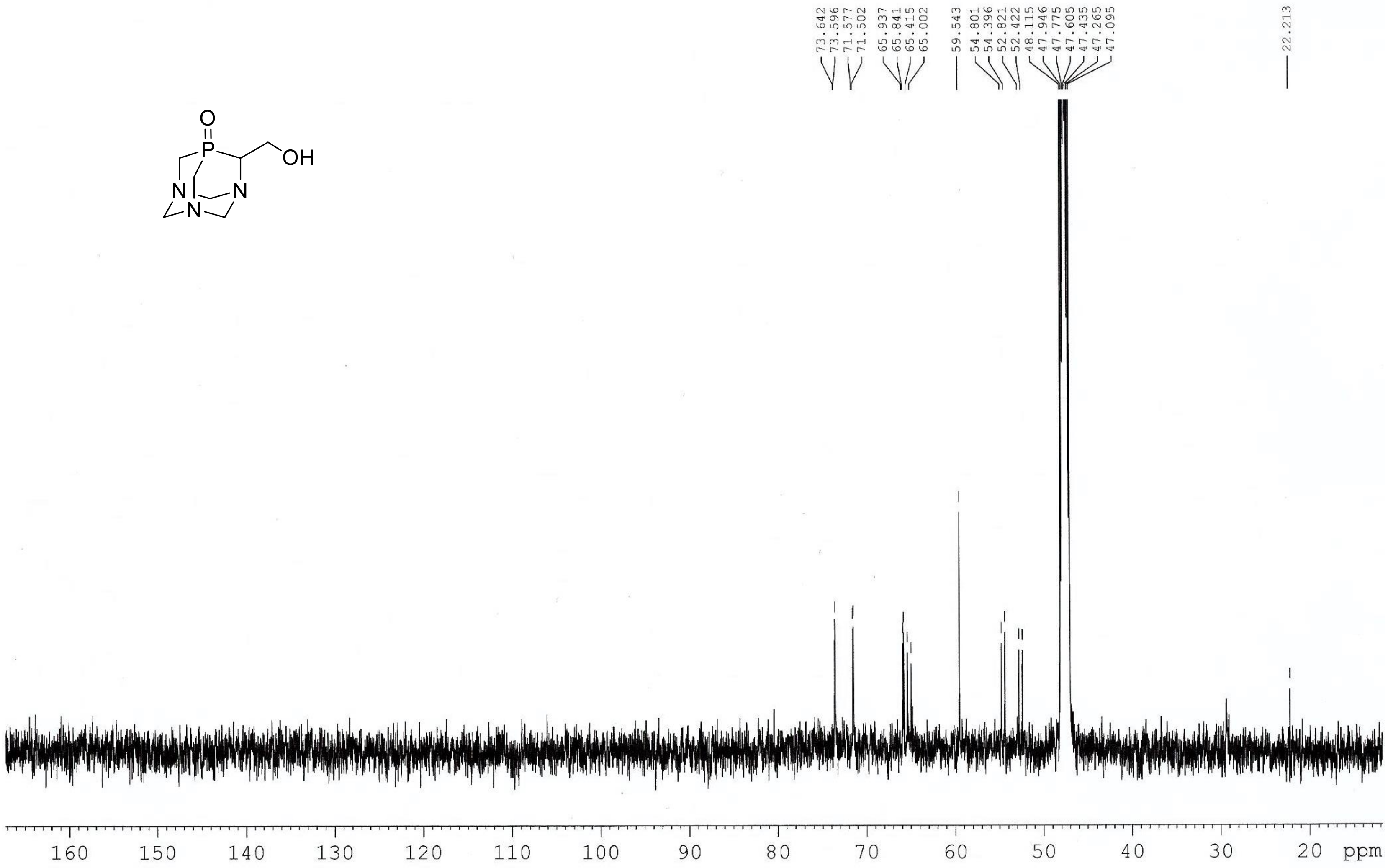

PTA(O)-CH_2OAc **11a**

$^{31}P\{^{1}H\}$ NMR (CD_3OD, 202 MHz)

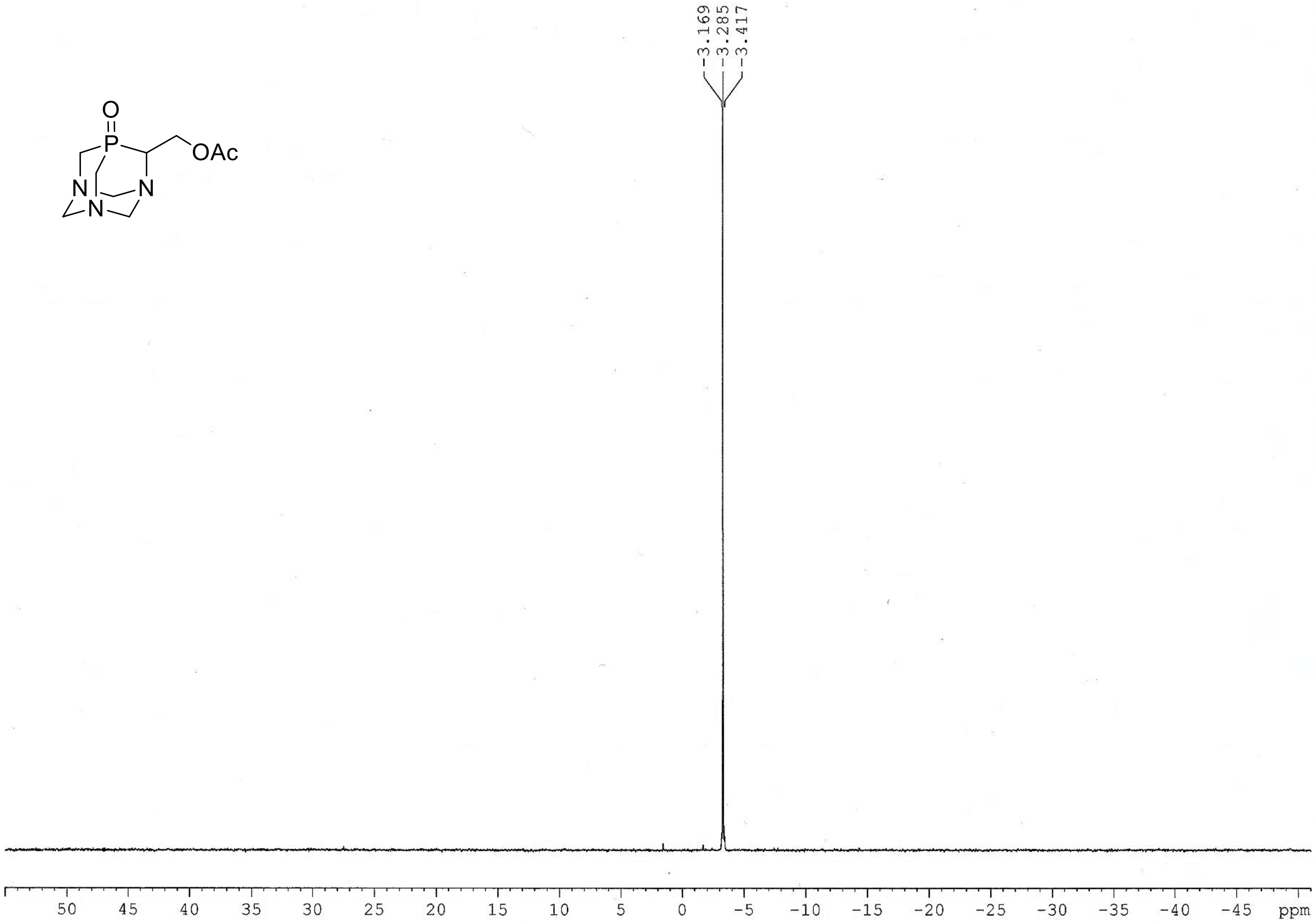

PTA(O)-CH$_2$OAc **11a**

^{1}H NMR (CD$_3$OD, 500 MHz)

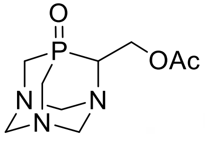

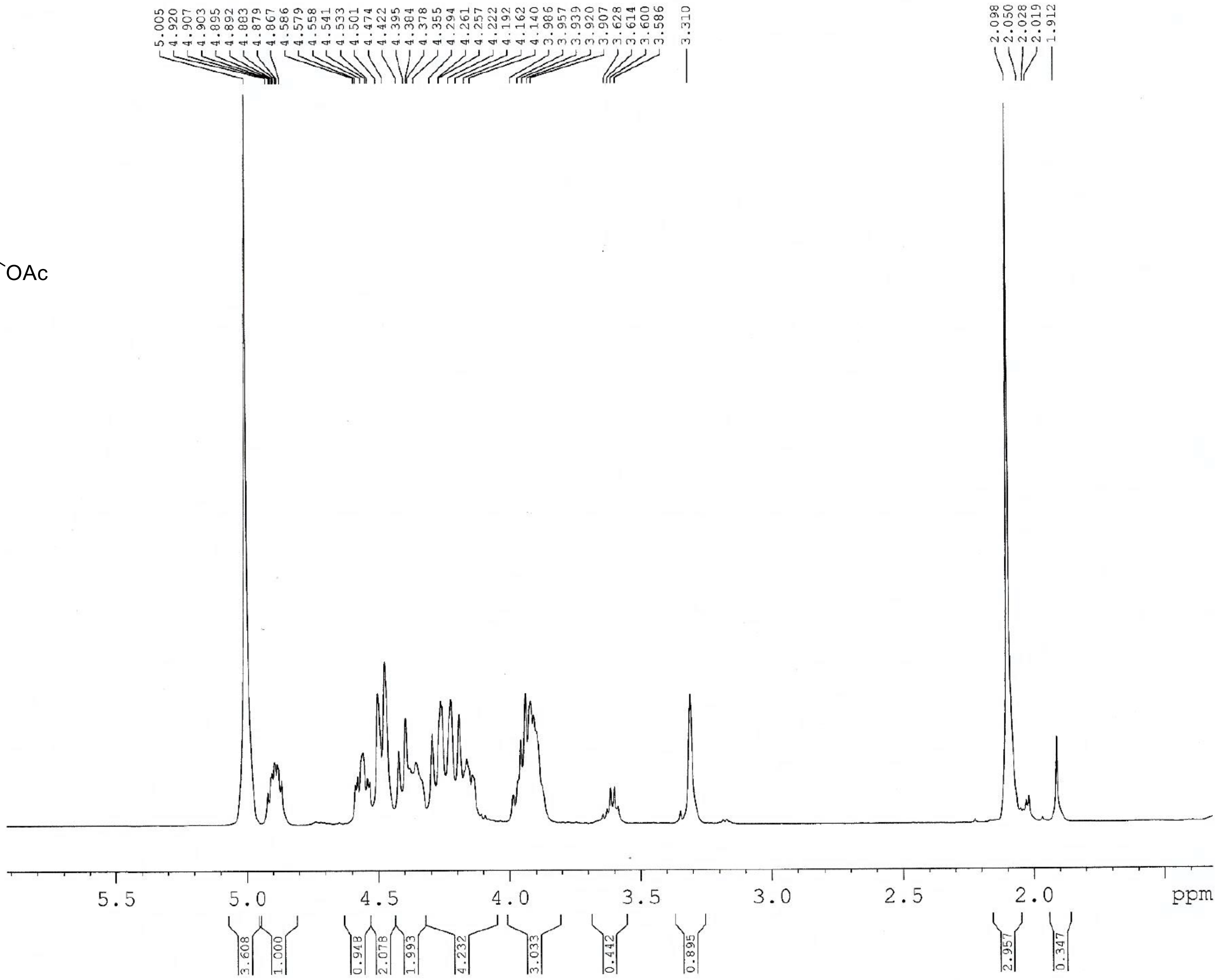

PTA(O)-CH_2OAc **11a**

$^{13}C\{^1H\}$ NMR (CD_3OD, 126 MHz)

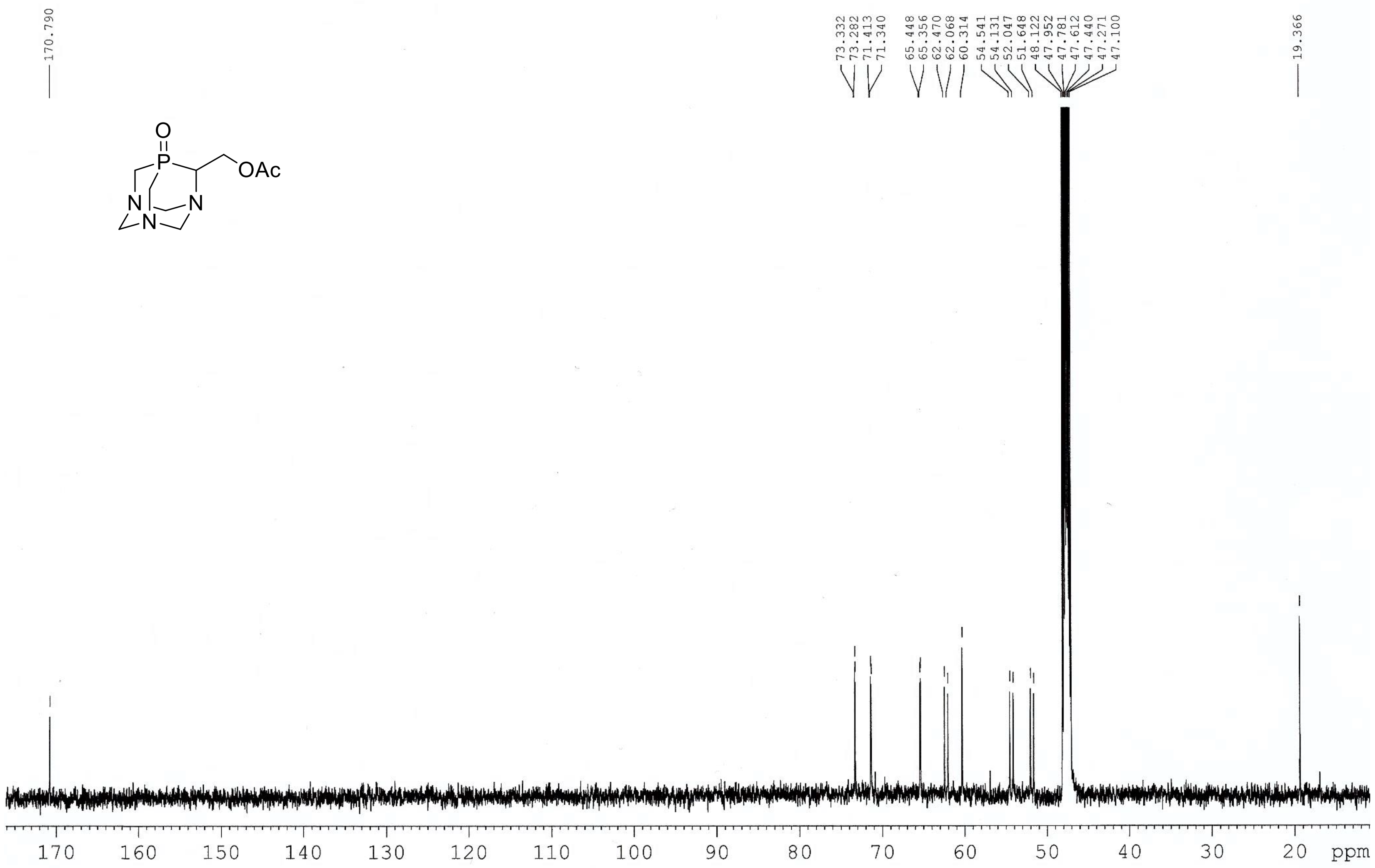

PTA(S)-CH_2OH **9a**

$^{31}P\{^1H\}$ NMR (CD_3OD, 81 MHz)

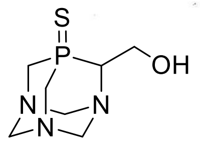

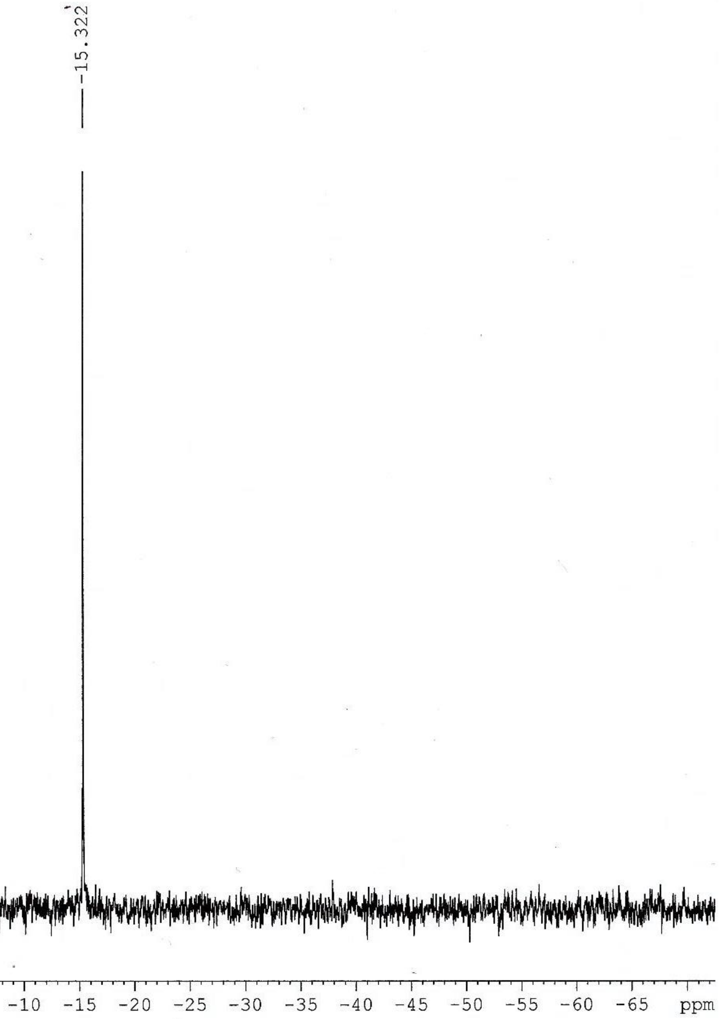

PTA(S)-CH_2OH **9a**

^1H NMR (CD_3OD, 500 MHz)

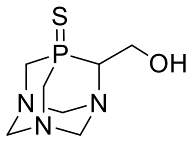

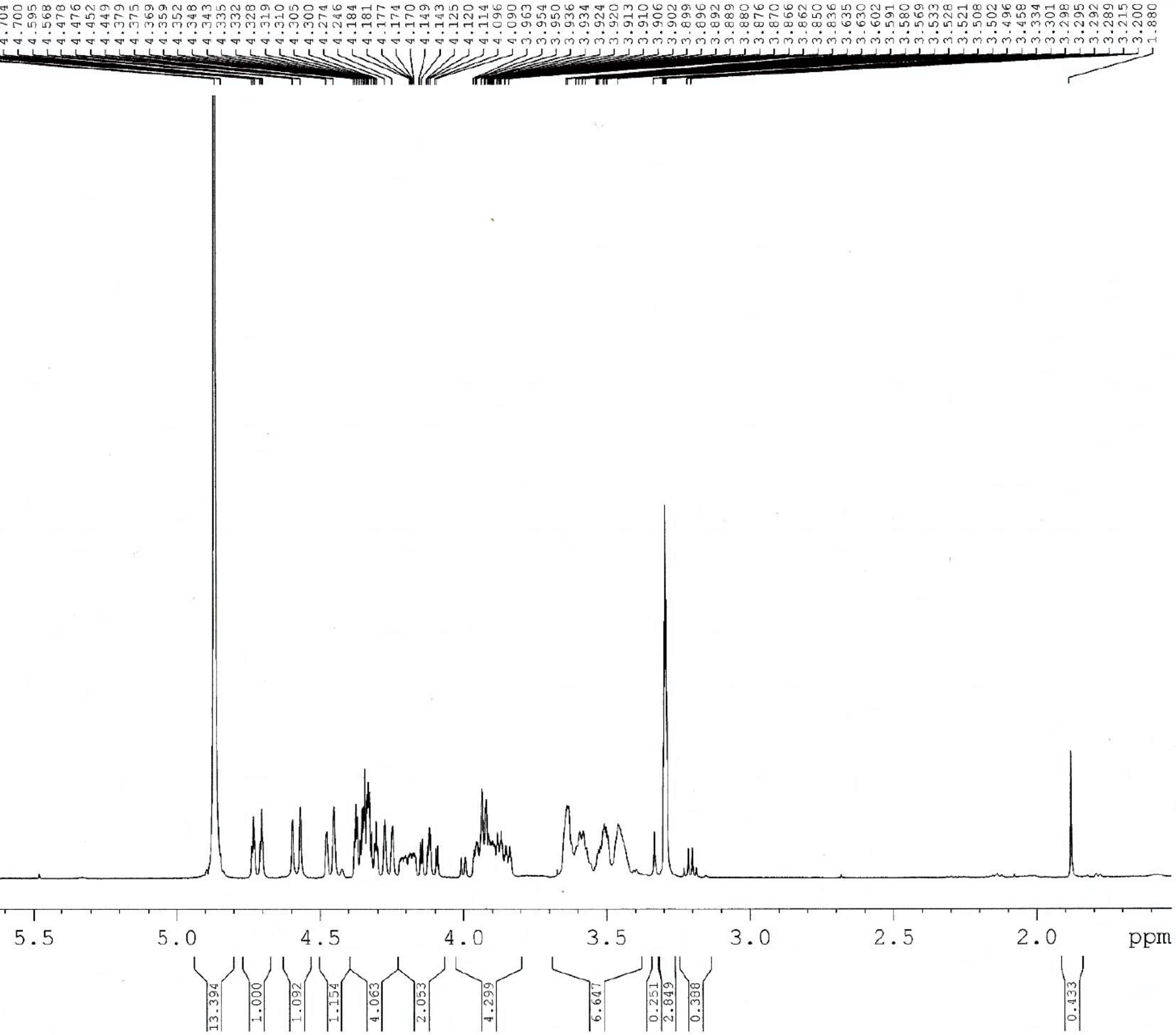

PTA(S)-CH$_2$OH **9a**

$^{13}C\{^1H\}$ NMR (CD_3OD, 126 MHz)

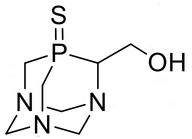

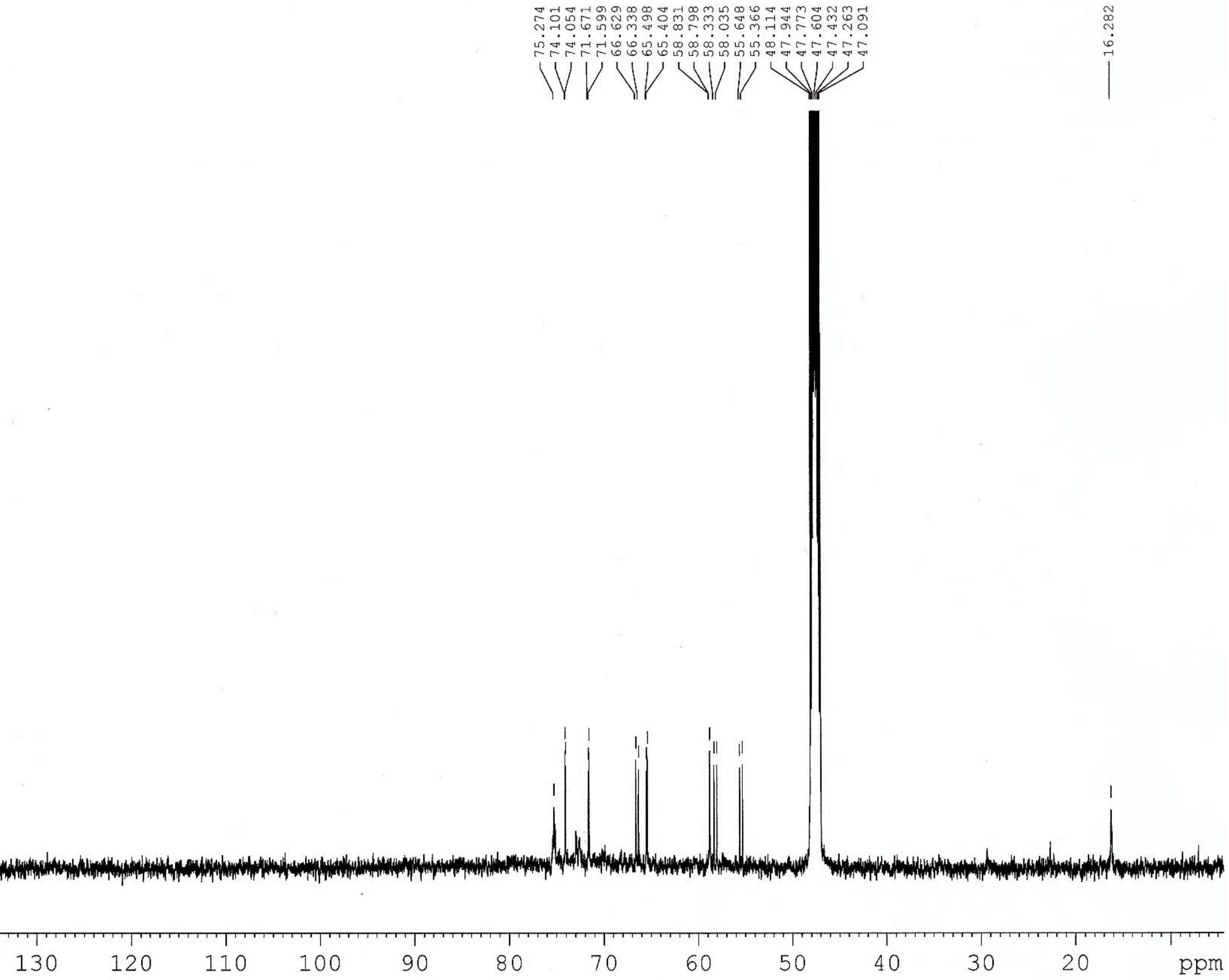

PTA(S)-CH_2OAc **12a**

$^{31}P\{^1H\}$ NMR ($CDCl_3$, 81 MHz)

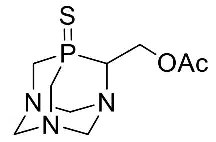

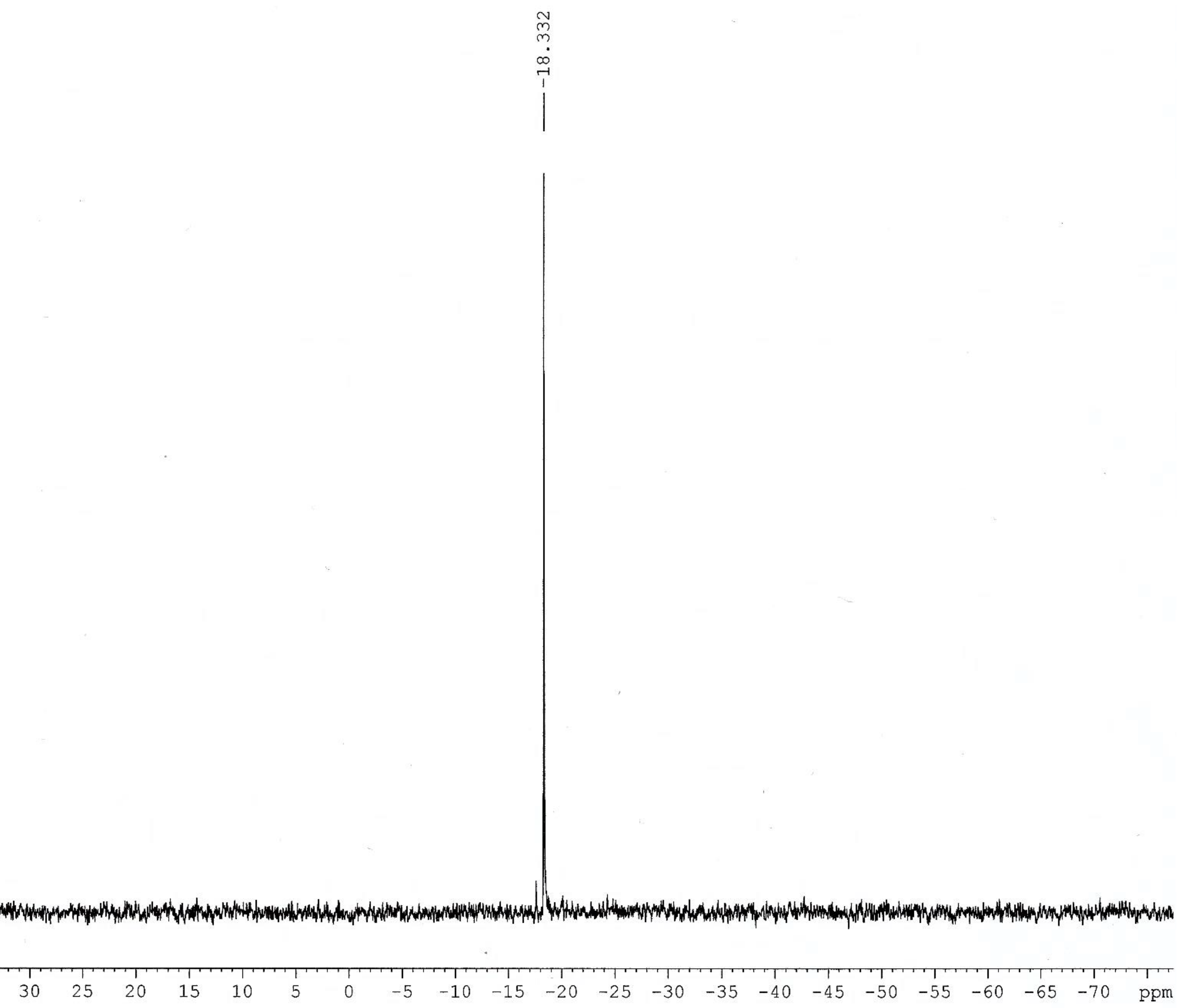

PTA(S)-CH_2OAc **12a**

^{1}H NMR (CD_3OD, 500 MHz)

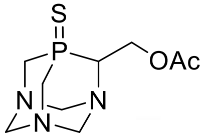

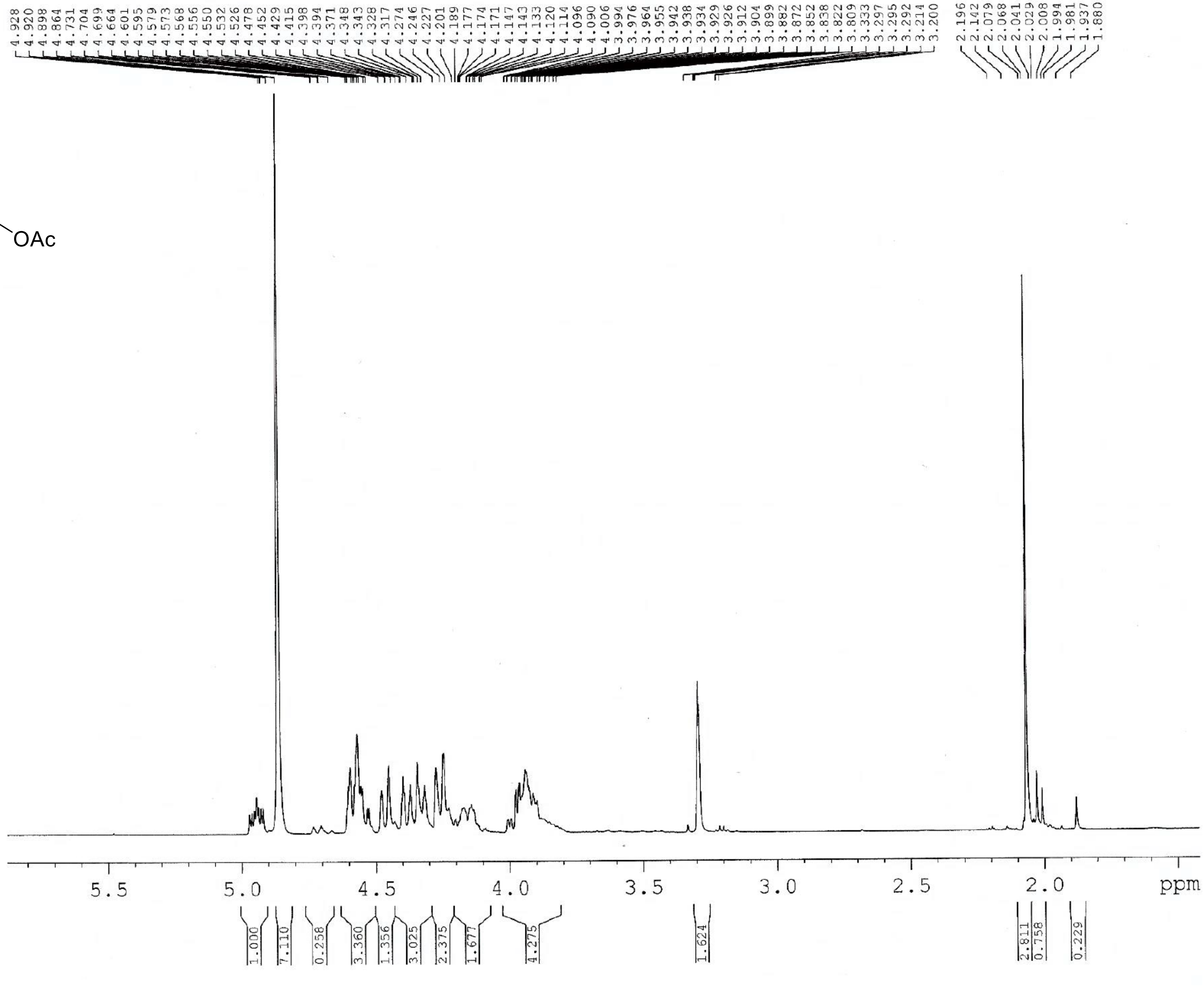

PTA(S)-CH_2OAc **12a**

$^{13}C\{^1H\}$ NMR (CD_3OD, 126 MHz)

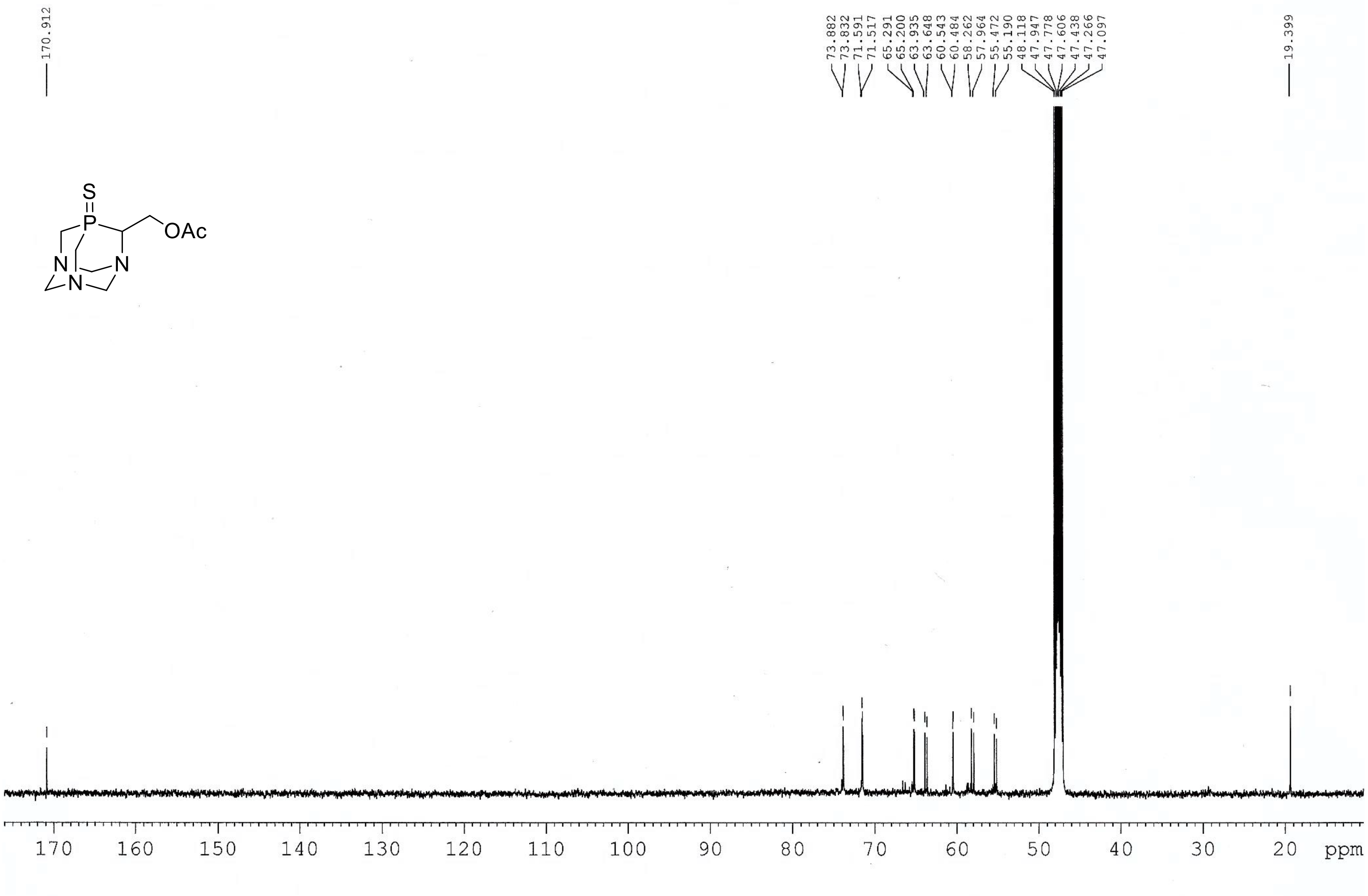

Supplement: Supplementary file 1 — jo0c02586_si_001.pdf [file jo0c02586_si_001.pdf]
